# The association of sleep duration with the risk of chronic kidney disease: a systematic review and meta-analysis

**DOI:** 10.1093/ckj/sfae177

**Published:** 2024-07-11

**Authors:** Jin Hean Koh, Brian Sheng Yep Yeo, Timothy Wei En Tan, Mark Yong Siang See, Adele Chin Wei Ng, Shaun Ray Han Loh, Joshua Gooley, Chieh Suai Tan, Song Tar Toh

**Affiliations:** Yong Loo Lin School of Medicine, National University of Singapore, Singapore, Singapore; Yong Loo Lin School of Medicine, National University of Singapore, Singapore, Singapore; Yong Loo Lin School of Medicine, National University of Singapore, Singapore, Singapore; Lee Kong Chian School of Medicine, Nanyang Technological University, Singapore, Singapore; Department of Otorhinolaryngology-Head and Neck Surgery, Singapore General Hospital, Singapore, Singapore; Department of Otorhinolaryngology-Head and Neck Surgery, Singapore General Hospital, Singapore, Singapore; Singhealth Duke-NUS Sleep Centre, Duke-NUS Medical School, Singapore, Singapore; Singhealth Duke-NUS Sleep Centre, Duke-NUS Medical School, Singapore, Singapore; Department of Renal Medicine, Singapore General Hospital, Singapore, Singapore; Department of Otorhinolaryngology-Head and Neck Surgery, Singapore General Hospital, Singapore, Singapore; Singhealth Duke-NUS Sleep Centre, Duke-NUS Medical School, Singapore, Singapore

**Keywords:** kidney failure, sleep hygiene, sleep quality

## Abstract

**Background and hypothesis:**

Published literature suggests that sleep duration and quality may be affected in adults with chronic kidney disease. However, the relationship between these two entities remains a matter of debate. The objective of this systematic review and meta-analysis is to assess the effect of sleep duration and quality on chronic kidney disease.

**Methods:**

A systematic review of the Medline/PubMed, Embase, Cochrane Library, and CINAHL databases was conducted for articles pertaining to the association between sleep duration and quality on chronic kidney disease. The main outcome was the hazard/risk ratio of chronic kidney disease in patients of varying sleep durations and quality.

**Results:**

In total, 42 studies (2 613 971 patients) with a mean age of 43.55 ± 14.01 years were included in the meta-analysis. Compared with a reference range of 7 to 8 hours of sleep, short sleep durations of ≤4 hours (RR 1.41, 95% CI: 1.16 to 1.71, *P *< 0.01), ≤5 hours (RR 1.46, 95% CI: 1.22 to 1.76, *P *< 0.01), ≤6 hours (RR 1.18, 95% CI: 1.09 to 1.29, *P *< 0.01), and ≤7 hours (RR 1.19, 95% CI: 1.12 to 1.28, *P *< 0.01) were significantly associated with an increased risk of incident chronic kidney disease. Long sleep durations of ≥8 hours (RR 1.15, 95% CI: 1.03 to 1.28, *P *< 0.01) and ≥9 hours (RR 1.46, 95% CI: 1.28 to 1.68, *P *< 0.01) were also significantly associated with an increased risk of incident chronic kidney disease. Meta-regression did not find any significant effect of age, gender, geographical region, and BMI and an association with sleep duration and risk of incident chronic kidney disease.

**Conclusion:**

Both short and long sleep durations were significantly associated with a higher risk of chronic kidney disease. Interventions targeted toward achieving an optimal duration of sleep may reduce the risk of incident chronic kidney disease.

KEY LEARNING POINTS **What was known**:

- Short and long sleep durations may be associated with an increased risk of chronic kidney disease, however, this relationship remains unclear.

**This study adds:**

- This analysis of pooled data sought to investigate the relationship between sleep duration and the risk of chronic kidney disease. From 39 studies including 2 525 312 patients, short and long sleep durations were associated with a higher risk of developing chronic kidney disease.

**Potential impact:**

- Interventions aimed at ensuring an optimal duration of sleep may be beneficial in reducing the risk of developing chronic kidney disease.

## INTRODUCTION

Chronic kidney disease (CKD) is a pervasive and escalating health issue affecting 10%–15% of the world's adult population [1]. Its prevalence continues to rise due to the widespread adoption of sedentary lifestyles and longer life expectancies resulting from improved medical knowledge and healthcare [2, 3]. The burden on human health and healthcare systems alike cannot be overstated, with an increasing number of patients suffering from severe complications such as magnified risks of cardiovascular disease, organ failure necessitating replacement therapy, and multi-factor mortality [4]. As the name suggests, this progressive condition traditionally develops as we age and over the course of many years, influenced by cumulative effects of both genetic and environmental factors, which range from abrupt disturbances in physiology and function, to more subtle and often overlooked parts of daily life [5]. Sleep is one such risk factor that has gradually risen as a contributor to not just kidney disease, but overall health as well, alongside other crucial factors such as diet and exercise [6].

The prevalence of sleep disturbances varies by demographics, but can affect up to 20%–40% of people worldwide [7]. In recent times, many studies have documented myriad downstream effects and associations of sleep duration and quality with various aspects of health [8]. The relationship between sleep and kidney disease has also garnered increasing attention, with the emergence of a few large cohort studies [9, 10]. In general, the association of shortened and prolonged sleep durations, as well as poor sleep quality, with CKD is supported by a substantial body of research [9, 10, 11]. However, there are some contradictory findings, and further objective clarity is needed to fully characterize this complex relationship, especially in different socio-cultural contexts [9, 11, 12].

The proposed mechanisms and pathophysiology underlying this relationship are diverse and many. Certain diseases, such as obstructive sleep apnea and other sleep disorders, can lead to disturbances in both sleep duration and quality. These are processes that are detrimental to kidney function such as sympathetic activation, nocturnal hypertension, hypoxia, oxidative stress, inflammation, and glomerular endothelial dysfunction [13, 14, 15]. Insufficient sleep, insomnia, and other disorders affecting circadian rhythm have also been linked to poor cognitive and emotional function, downstream hormone imbalances such as hypercortisolism, and other risk factors for CKD such as aspects of metabolic syndrome [16]. Furthermore, CKD and declining renal function itself can also impact sleep, with an increased prevalence of restless leg syndrome and other potential effects of uremia and other deranged electrolytes, adding to the complexity of CKD management [12, 17]. Patients on renal replacement therapy often report poorer sleep quality compared with healthy controls, along with poorer sleep efficiency and fragmentation index [18]. Various modalities of renal replacement therapy have their treatment-related burdens for the patient, which may affect their sleep. Apart from anemia and fluid and toxin accumulation, patients can also suffer from pain, pruritus, and psychological problems [19, 20].

In summary, CKD and sleep are intricate and interrelated conditions that require integrated management to optimal outcomes in patients [9, 11, 12]. However, the contributing value of poor sleep to the increasing incidence of CKD, and therefore its role in the prevention and management of CKD remains unclear [9, 10, 12]. In a world on the cusp of a paradigm shift toward preventive medicine, the understanding and education of upstream risk factors must improve, and the need to optimize an aspect as fundamental and integral to human life as sleep remains a topmost priority and, indeed, necessity [9]. This systematic review and meta-analysis aims to provide an updated overview of the current body of knowledge around the relationship between sleep and the risk of CKD, with the hypothesis that there is a significant impact of poor sleep on the risk of CKD, which merely awaits objective characterization for appropriate intervention. The authors believe this study will provide much-needed clarity and guidance on this important frontier of health, driving progress toward improved CKD prevention and management strategies.

## MATERIALS AND METHODS

### Data sources and searches

The protocol for this review was registered with PROSPERO (CRD42023420030). With reference to the Preferred Reporting Items for Systematic Review and Meta-Analyses (PRISMA) guidelines, a search was conducted on Medline/PubMed, Embase, the Cochrane Register for Controlled Trials (CENTRAL), and the Cumulative Index of Nursing and Allied Health (CINAHL) databases for studies published from inception to 19 June 2023 [21]. The search strategy used a combination of free text words and medical subject heading terms (Supplement 1). The reference lists of systematic reviews and included articles and the gray literature were also screened manually to identify additional studies for a comprehensive search. Contact with authors of included studies was made where feasible to collect supplementary data.

### Study selection

Two blinded authors (M.S. and T.T.) independently screened titles and abstracts to check the eligibility for inclusion using the online platform Rayyan, with disputes being resolved through consensus from a third independent author (J.H.K.) [22]. Reviewers then assessed the full texts of shortlisted studies against the following pre-defined inclusion and exclusion criteria.

The inclusion criteria were (i) observational studies that investigated the association between sleep duration and CKD in participants aged 18 years or older, (ii) full-text studies, (iii) studies published in a peer-reviewed journal, and (iv) studies published in the English language. Participants with CKD were defined as individuals with an estimated glomerular filtration rate (eGFR) <60 ml/min/1.73 m^2^ over 3 months or more, irrespective of cause, in accordance with the published definitions of the National Kidney Foundation Kidney Disease Outcomes Quality Initiative (KDIQI) [23]. For the outcome of incident CKD, studies were eligible if they included patients without a diagnosis of CKD, not on hemodialysis or peritoneal dialysis, or not receiving renal transplant at baseline. For the outcome of prevalent CKD, studies were eligible if they included patients who had a diagnosis of CKD, not on hemodialysis or peritoneal dialysis, or not receiving renal transplant at baseline. The included studies employed subjective and objective sleep measurements, which comprised subjective sleep questionnaires such as the Pittsburgh Sleep Quality Index (PSQI), and objective sleep measurements such as polysomnography.

The exclusion criteria were (i) studies including participants <18 years old, (ii) animal studies, (iii) case reports, (iv) *in vitro* studies, and (v) reviews.

### Data collection

Data from included articles were collected by two blinded, independent reviewers (M.S. and T.T.) in duplicate onto a structured proforma specifically designed for the study and piloted beforehand on a sample of selected studies. Disagreement was resolved by discussion and consensus with a third reviewer (J.H.K.). The data collection sheet contained key characteristics of studies, according to the Population, Intervention, Comparison, Outcome, Study Type framework [24, 25]. Data collected included geographical region; sample size, inclusion and exclusion criteria; baseline characteristics of participants such as mean age, gender, ethnicity, and mean BMI; comorbidities such as diabetes, hypertension, hyperlipidemia, coronary artery disease, and smoking status CKD definition; details of how CKD was assessed; stage of CKD stage; summary measures of incident; and prevalent CKD across various sleep durations and sleep quality as measured with the PSQI, with corresponding 95% confidence intervals, publication year, year of study completion, and study design. The PSQI is a self-rated questionnaire that assesses sleep quality and disturbances over a 1-month time interval, with seven components involving subjective sleep quality, sleep latency, sleep duration, habitual sleep efficiency, sleep disturbances, use of sleeping medication, and daytime dysfunction [26].

### Risk of bias assessment

Two blinded, independent reviewers (M.S. and T.T.) conducted the risk of bias assessment of included articles using the Risk of Bias in Non-randomized Studies of Exposures (ROBINS-E) tool. The ROBINS-E is designed specifically to assess the risk of bias of cohort studies on the premises of confounding, measurement of exposure, selection of participants, post-exposure interventions, missing data, measurement of outcome, and selection of the reported result [27]. Exclusion of low-quality studies was performed only during sensitivity analyses in an objective to explore the result's heterogeneity. Otherwise, all studies were retained independently of their quality, following Glass's approach [28].

### Publication bias

Publication bias was assessed by visual inspection of the respective funnel plots [29]. The asymmetry of funnel plots was further assessed using Egger's linear regression method and Begg's test [30]. Missing studies were imputed using the trim-and-fill method [31].

### Statistical analysis

All analysis was conducted in R Studio (version 4.2.2) using the meta package [32]. Descriptive statistics were presented as means and standard deviations for continuous variables and counts for categorical variables. When studies reported medians and interquartile ranges, these were converted to means and standard deviations using the methods of Wan *et al.* [33] A conventional pairwise meta-analysis was done in risk ratios. The meta-analysis compared CKD risk for short (<7 hours) and long (>8 hours), versus referent sleep duration (mostly 7 to 8 hours). Where the baseline category differed from 7 to 8 hours, the baseline category was switched using the generalized least squares method [34]. In studies where multiple categories of short or long sleep were available, a single estimate was obtained using the generalized least squares method [34]. Random-effects models were used in all analysis regardless of heterogeneity as recent evidence suggests that it provides more robust outcome measures compared to the alternative fixed effects models [35]. The Hartung–Knapp method was also implemented to adjust the confidence interval of the overall estimate [36].

Statistical heterogeneity was assessed via *I*^2^ and Cochran Q test values, where an *I*^2^ value of <25% represented low heterogeneity and an *I*^2^ value ≥25% represented moderate to high heterogeneity [37, 38]. A Cochran *Q* test with a *P* value of ≤0.10 was considered significant for heterogeneity.

Where 10 or more studies were available for a particular outcome, additional analyses were conducted to evaluate potential sources of heterogeneity between studies [39]. Apart from subgroup analyses, univariate random-effects meta-regression were conducted, and effect moderators were confirmed using permutation testing with 1000 iterations to eliminate spurious results [40, 41]. Statistical significance was considered for outcomes with a *P* ≤ 0.05. Leave-out-one influence analyses were performed to examine the influence of individual studies on the overall findings. Cumulative meta-analyses were performed ranked by year published, to examine the stability of published data over time.

### Certainty of evidence

The quality of pooled evidence was evaluated using the Grading of Recommendations Assessment, Development and Evaluations (GRADE) framework [42]. The GRADE framework rates each study on the basis of study design, consistency, directness, risk of bias, precision, and publication bias. For each outcome, the level of evidence was rated as high, moderate, low, or very low.

## RESULTS

### Literature search

**Figure 1:**
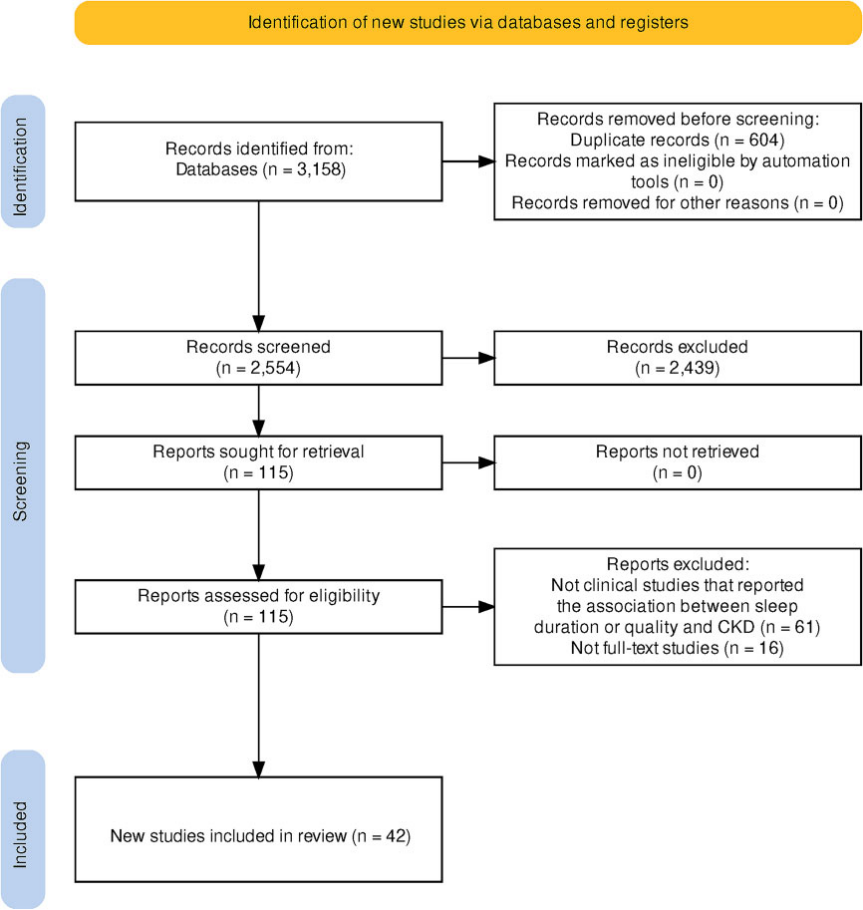
PRISMA flow diagram.

In the initial search, 3158 articles were included after removal of duplicates, of which 115 were selected for full-text review: 42 articles met the final inclusion criteria [9–11, 12, 43–79, 80], 21 studies were prospective cohort studies, and 21 studies were cross-sectional studies. The inter-rate reliability as assessed by Cohen's kappa was 0.98 [81]. Figure 1 shows the PRISMA flow diagram that summarizes the study selection process.

### Study characteristics

The total sample size was 2 613 971 participants. The mean age of the participants was 43.55 ± 14.01 years, and 73% of patients were male. The included studies were completed between 1998 and 2020. Three studies measured sleep duration and quality by polysomnography or actigraphy, whereas 39 studies measured sleep duration and quality by self-reported sleep questionnaire. Table 1 and 2 contains the summary of the key characteristics for included articles. Of the 43 included studies, 25 were rated at low risk of bias, 10 at moderate risk of bias, and seven at high risk of bias (Supplement 2).

**Table 1:** Characteristics of included studies.

Author	Study design	Inclusion criteria	Geographical region	Stage of CKD	Method of diagnosis of CKD	Method of measurement of sleep duration and quality
Agarwal *et al.* [12]	Cohort study	Patients with CKD or on hemodialysis	North America	3 to 5	eGFR measurements	Actigraphy
Bo *et al.* [9]	Cohort study	Patients from a standard medical examination program conducted by the MJ Health Management Institution (Taipei, Taiwan) from 1996 to 2014	Asia	3 to 5	eGFR measurements	Self-reported questionnaire
Cha *et al.* [11]	Cohort study	Patients from the KoGES Study	Asia	3 to 5	eGFR measurements	Self-reported questionnaire
Chang *et al.* [10]	Cohort study	Patients from the KHANES Survey over 19 years old with information on sleep parameters	Asia	3 to 5	eGFR measurements	Self-reported questionnaire
Choi *et al.* [43]	Cross-sectional study	Patients from the KoGES study	Asia	3 to 5	eGFR measurements	Self-reported questionnaire
Cohen *et al.* [44]	Cross-sectional study	Predialysis patients with CKD	North America	3 to 5	eGFR measurements	Self-reported questionnaire
Del Brutto *et al.* [45]	Cross-sectional study	Atahualpa residents aged ≥60 years who were offered a brain MRI	South America	3 to 5	eGFR measurements	Self-reported questionnaire
Fang *et al.* [46]	Cross-sectional study	Patients from the KHANES Survey	Asia	3 to 5	eGFR measurements	Self-reported questionnaire
Fujibayashi *et al.* [47]	Cross-sectional study	Patients who underwent annual health checkups between May 2006 to August 2010	Asia	3 to 4	eGFR measurements	Self-reported questionnaire
Geng *et al.* [48]	Cohort study	Patients from the Singapore Chinese Health Study with information on daily sleep duration	Asia	5	eGFR measurements	Self-reported questionnaire
Gu *et al.* [49]	Cohort study	Adult Patients from the KHANES with type 2 diabetes mellitus	Asia	3 to 5	eGFR measurements	Self-reported questionnaire
Hirano *et al.* [50]	Cohort study	Patients from the St Luke's International Hospital in Tokyo	Asia	3 to 5	eGFR measurements	Self-reported questionnaire
Jean-Lous *et al.* [51]	Cross-sectional study	Patients from the National Health Interview Survey	North America	3 to 5	eGFR measurements	Self-reported questionnaire
Jiang *et al.* [52]	Cohort study	Patients from the China Health and Retirement Longitudinal Study	Asia	3 to 5	Self-reported	Self-reported questionnaire
Karatas *et al.* [53]	Cohort study	Patients who were predialysis or undergoing hemodialysis	Africa	3 to 5	eGFR measurements	Self-reported questionnaire
Kim *et al.* [54]	Cohort study	Patients from the Kangbuk Samsung Health Study	Asia	3 to 5	eGFR measurements	Self-reported questionnaire
Kucuk *et al.* [55]	Cohort study	Patients with CKD, undergoing hemodialysis, peritoneal dialysis, or post-renal transplant	Asia	4 to 5	eGFR measurements	Self-reported questionnaire
Lee *et al.* [56]	Cohort study	Patients with CKD or ESRD	North America	3 to 5	eGFR measurements	Self-reported questionnaire
Li *et al.* [57]	Cross-sectional study	Patients from the Kailuan cohort study	Asia	3 to 5	eGFR measurements	Self-reported questionnaire
Lu *et al.* [58]	Cross-sectional study	Patients from the 2013, 2014 and 2016 Behavioral Risk Factor Surveillance System	North America	3 to 5	Self-reported	Self-reported questionnaire
Meng *et al.* [59]	Cross-sectional study	Cross-sectional study from the Tianjin Metabolic Diseases Hospital	Asia	3 to 5	eGFR measurements	Self-reported questionnaire
Nagai *et al.* [60]	Cross-sectional study	Patients from the Jichi Medical School Ambulatory Blood Pressure Monitoring study	Asia	3 to 5	eGFR measurements	Self-reported questionnaire
Nakajima *et al.* [61]	Cohort study	Patients from the NAFLD in Gifu Area, Longitudinal Analysis study	Asia	3 to 5	eGFR measurements	Self-reported questionnaire
Nishimura *et al.* [62]	Cohort study	Patients with type 2 diabetes	Asia	3 to 5	eGFR measurements	Polysomnography
Petrov *et al.* [63]	Cohort study	Patients from the 2009–2012 National Health and Nutrition Examination Survey	Asia	1 to 5	eGFR measurements	Self-reported questionnaire
Pinto *et al.* [64]	Cross-sectional study	Patients with CKD compared with healthy controls	Asia	5	eGFR measurements	Self-reported questionnaire
Platinga *et al.* [65]	Cross-sectional study	Patients from the National Health and Nutrition Examination Survey 2005–2008	North America	3 to 4	eGFR measurements	Self-reported questionnaire
Sabbatini *et al.* [66]	Cross-sectional study	Kidney graft recipients and patients on hemodialysis, compared with healthy controls	South America	5	eGFR measurements	Self-reported questionnaire
Salifu *et al.* [67]	Cross-sectional study	Cross-sectional survey of the National Health Interview Survey	North America	3 to 5	Self-reported	Self-reported questionnaire
Sasaki *et al* [68]	Cross-sectional study	Patients who underwent annual health checkups between April 2003 and March 2004	North America	3 to 5	eGFR measurements	Self-reported questionnaire
Sun *et al.* [69]	Cross-sectional study	Patients from the 2011 and 2015 surveys of the CHARLS	Europe	3 to 5	eGFR measurements	Self-reported questionnaire
Tan *et al.* [70]	Cross-sectional study	Patients from the Singapore Malay Eye Study and Singapore Indian Eye Study	North America	3 to 5	eGFR measurements	Self-reported questionnaire
Tu *et al.* [71]	Cohort study	Patients with CKD not on dialysis	Asia	3 to 5	eGFR measurements	Self-reported questionnaire
Unruh *et al.* [72]	Cohort study	Patients undergoing hemodialysis, compared with healthy controls	Asia	5	eGFR measurements	Polysomnography
Wang *et al.* [73]	Cross-sectional study	Patients from CHARLS	Asia	3 to 5	Self-reported	Self-reported questionnaire
Warsame *et al.* [74]	Cross-sectional study	Patients from the NHANES cohort aged ≥60 years of age with measured serum creatine and at least one assessment of cognitive function	Asia	3 to 5	eGFR measurements	Self-reported questionnaire
Wu *et al.* [75]	Cross-sectional study	Participants from the NHANES	North America	3 to 5	eGFR measurements	Self-reported questionnaire
Xu *et al.* [76]	Cohort study	Patients from the CHARLS study	Asia	3 to 5	eGFR measurements	Self-reported questionnaire
Yamamoto *et al.* [77]	Cohort study	Patients who underwent annual health checkups between April 2006 and 2010	Asia	3 to 5	eGFR measurements	Self-reported questionnaire
Ye *et al.* [78]	Cross-sectional study	Patients from the risk evaluation of cancers in Chinese diabetic individuals: a longitudinal (REACTION) study	North America	3 to 5	eGFR measurements	Self-reported questionnaire
Yu *et al.* [79]	Cross-sectional study	Patients from the KHANES >19 years old with information on sleep parameters	North America	3 to 5	eGFR measurements	Self-reported questionnaire
Zhang *et al.* [80]	Cohort study	Patients from the UK Biobank	Asia	3 to 5	eGFR measurements	Self-reported questionnaire

CHARLS, China Health and Retirement Longitudinal Study; DM, diabetes mellitus; HTN, hypertension; HLD, hyperlipidemia; KoGES, Korea Genome and Epidemiology Study; KHANES, Korea National Health and Nutritional Examination; NHANES, National Health and Nutrition Examination Survey; NR, not reported

**Table 2:** Characteristics of included patients.

Author	Total population	Number with CKD	Mean age (years)	Male (%)	Mean BMI (kg/m^2^)	Obesity (%)	DM (%)	HTN (%)	HLD (%)	CAD (%)	Smoking (%)
Agarwal *et al.* [12]	280	261	61.10	83	29.2	NR	47.00	NR	NR	NR	NR
Bo *et al.* [9]	194 039	8519	38.20	51	22.8	NR	NR	NR	NR	0.00	23.00
Cha *et al.* [11]	2837	642	55.40	50	NR	31.00	34.91	11.66	NR	0.00	40.70
Chang *et al.* [10]	26 249	963	39.30	55	22.1	24.80	3.20	13.38	11.05	NR	NR
Choi *et al*. [43]	1360	125	60.00	40	24.5	NR	12.10	37.90	24.00	7.60	11.30
Cohen *et al.* [44]	153	92	61.50	51	NR	NR	46.10	NR	NR	NR	NR
Del Brutto *et al.* [45]	314	182	71.10	31	NR	NR	NR	NR	NR	NR	NR
Fang *et al.* [46]	17 418	963	51.30	44	23.9	NR	24.50	24.50	NR	NR	39.48
Fujibayashi *et al.* [47]	25 493	2748	48.50	74	23.1	24.90	NR	NR	NR	2.20	55.20
Geng *et al.* [48]	63 147	1143	56.40	44	23.1	NR	8.90	23.60	NR	0.00	31.30
Gu *et al.* [49]	5930	742	58.70	49	24.8	NR	100.00	60.00	29.80	0.00	48.10
Hirano *et al.* [50]	76 705	4214	45.80	49	NR	19.20	2.00	7.70	4.60	0.00	38.70
Jean-Lous *et al.* [51]	123 967	305	42.00	50	NR	27.70	8.20	24.10	NR	3.00	42.40
Jiang *et al.* [52]	11 677	560	58.70	45	23.4	NR	NR	NR	NR	0.00	69.90
Karatas *et al.* [53]	240	149	52.80	52	NR	NR	NR	NR	NR	NR	NR
Kim *et al.* [54]	241 607	644	40.60	56	24.5	39.40	6.10	16.50	NR	1.60	61.40
Kucuk *et al.* [55]	140	140	43.00	51	NR	NR	NR	NR	NR	NR	NR
Lee *et al.* [56]	500	500	60.00	60	28.6	NR	8.66	50.40	NR	55.10	NR
Li *et al.* [57]	11 040	425	53.70	NR	25.0	NR	26.00	67.30	NR	1.35	44.20
Lu *et al.* [58]	1 191 768	40 866	NR	51	NR	NR	NR	NR	NR	NR	NR
Meng *et al.* [59]	336	102	52.70	53	26.7	NR	NR	NR	NR	NR	37.00
Nagai *et al.* [60]	514	194	72.30	37	24.6	NR	14.70	NR	21.10	0.00	21.30
Nakajima *et al.* [61]	2201	2201	42.10	54	22.0	NR	NR	NR	NR	0.00	63.00
Nishimura *et al.* [62]	462	303	58.50	86	27.0	NR	100.00	68.60	NR	NR	23.40
Petrov *et al.* [63]	8030	601	46.40	51	NR	33.90	7.70	31.00	NR	3.10	NR
Pinto *et al.* [64]	39	20	54.90	NR	NR	NR	NR	NR	NR	NR	NR
Platinga *et al.* [65]	9110	1805	47.20	50	NR	33.80	8.30	42.00	NR	8.50	19.90
Sabbatini *et al.* [66]	715	546	40.80	60	23.6	NR	NR	NR	NR	NR	NR
Salifu *et al.* [67]	128 479	2480	59.60	45	NR	36.70	34.20	68.00	NR	22.60	54.50
Sasaki *et al.* [68]	3600	182	47.00	78	23.1	NR	0.00	0.00	0.00	0.00	63.40
Sun *et al.* [69]	11 339	887	58.80	47	23.8	10.72	11.60	37.90	NR	NR	39.28
Tan *et al.* [70]	1258	268	64.60	49	27.4	67.50	100.00	91.50	NR	20.50	11.10
Tu *et al.* [71]	326	326	57.87	73	26.5	NR	53.60	69.70	NR	NR	16.60
Unruh *et al.* [72]	184	46	62.70	72	28.0	NR	32.60	NR	NR	32.60	56.50
Wang *et al.* [73]	4003	NR	52.48	43	24.1	NR	5.30	18.70	NR	8.20	36.60
Warsame *et al.* [74]	3215	1061	NR	46	27.9	NR	23.10	75.40	NR	8.80	50.10
Wu *et al.* [75]	8269	223	48.50	49	30.0	NR	13.80	37.90	32.20	5.74	NR
Xu *et al.* [76]	4188	346	58.90	43	23.7	NR	8.00	NR	NR	12.20	28.60
Yamamoto *et al.* [77]	6834	550	34.00	50	22.0	NR	0.50	2.40	1.70	0.30	19.10
Ye *et al.* [78]	33 850	NR	58.10	33	24.7	15.10	22.80	60.30	NR	NR	15.20
Yu *et al.* [79]	19 994	NR	52.50	52	24.0	NR	7.00	1.10	8.00	NR	0.90
Zhang *et al.* [80]	370 671	6365	56.40	45	29.2	NR	8.90	NR	NR	NR	45.80

DM, diabetes mellitus; HTN, hypertension; HLD, hyperlipidemia; NR, not reported

### Incident CKD

#### Meta-analysis for short sleep duration

**Figure 2:**
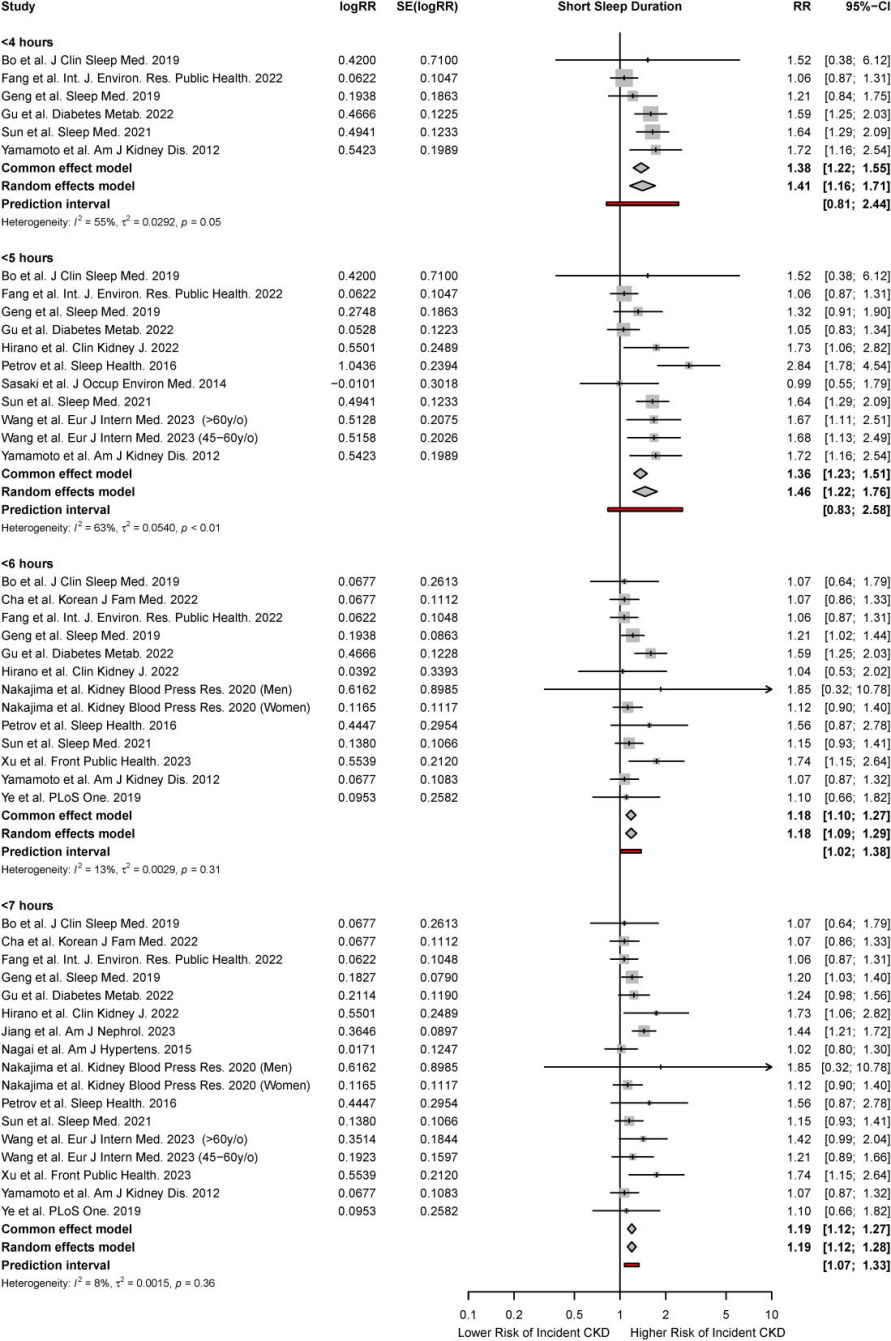
Forest plot for association of short sleep duration with a risk of incident CKD.

There were 14 studies that reported the association of short sleep duration with the risk of incident CKD [9, 11, 46, 48, 49, 50, 52, 60, 61, 63, 69, 76, 77, 78]. Based on the random-effects model, short sleep durations of ≤4 hours (HR, 1.41; 95% CI, 1.16 to 1.71; *P* < 0.01, *I*^2^* = *55%), ≤5 hours (HR, 1.46; 95% CI, 1.22 to 1.76; *P* < 0.01, *I*^2^* = *63%), ≤6 hours (HR, 1.18; 95% CI, 1.09 to 1.29; *P* < 0.01, *I*^2^* = *13%), and ≤7 hours (HR, 1.19; 95% CI, 1.12 to 1.28; *P* < 0.01, *I*^2^* = *8%) were associated with a significantly increased risk of incident CKD. Significant associations were observed across both fixed- and random-effects models (Fig. 2).

Given that the meta-analysis of short sleep duration and risk of incident CKD contained sufficient studies for further analyses, meta-regression was also performed to examine the influence of study-level covariates on the risk of incident CKD. Meta-regression found that the percentage of male gender was a statistically significant effect moderator of the association of sleep duration ≤4 hours and risk of incident CKD, accounting for 67.22% of heterogeneity and leaving 43.72% of residual heterogeneity. The pooled RR increased by a factor of 0.97 (95% CI, 0.60 to 4.41) per 1% increase in number of male participants. The bubble plot is shown in Supplement 3. Other characteristics including mean age, year of study completion, percentage of male participants, percentage of obese participants, and mean BMI were not significant effect moderators of the risk of incident CKD for the other classifications of sleep duration (Supplement 4).

For all outcomes, although visual inspection suggested the possibility of funnel plot asymmetry, this was not shown by Egger's test. Trim and fill resulted in minimal change to the pooled effect sizes. For all analyses, leave-one-out influence analysis showed that no single study had a drastic change on the pooled risk ratio, and cumulative meta-analysis showed a significant and stable pooled effect size (Supplements 5 and 6).

#### Meta-analysis for long sleep duration

**Figure 3:**
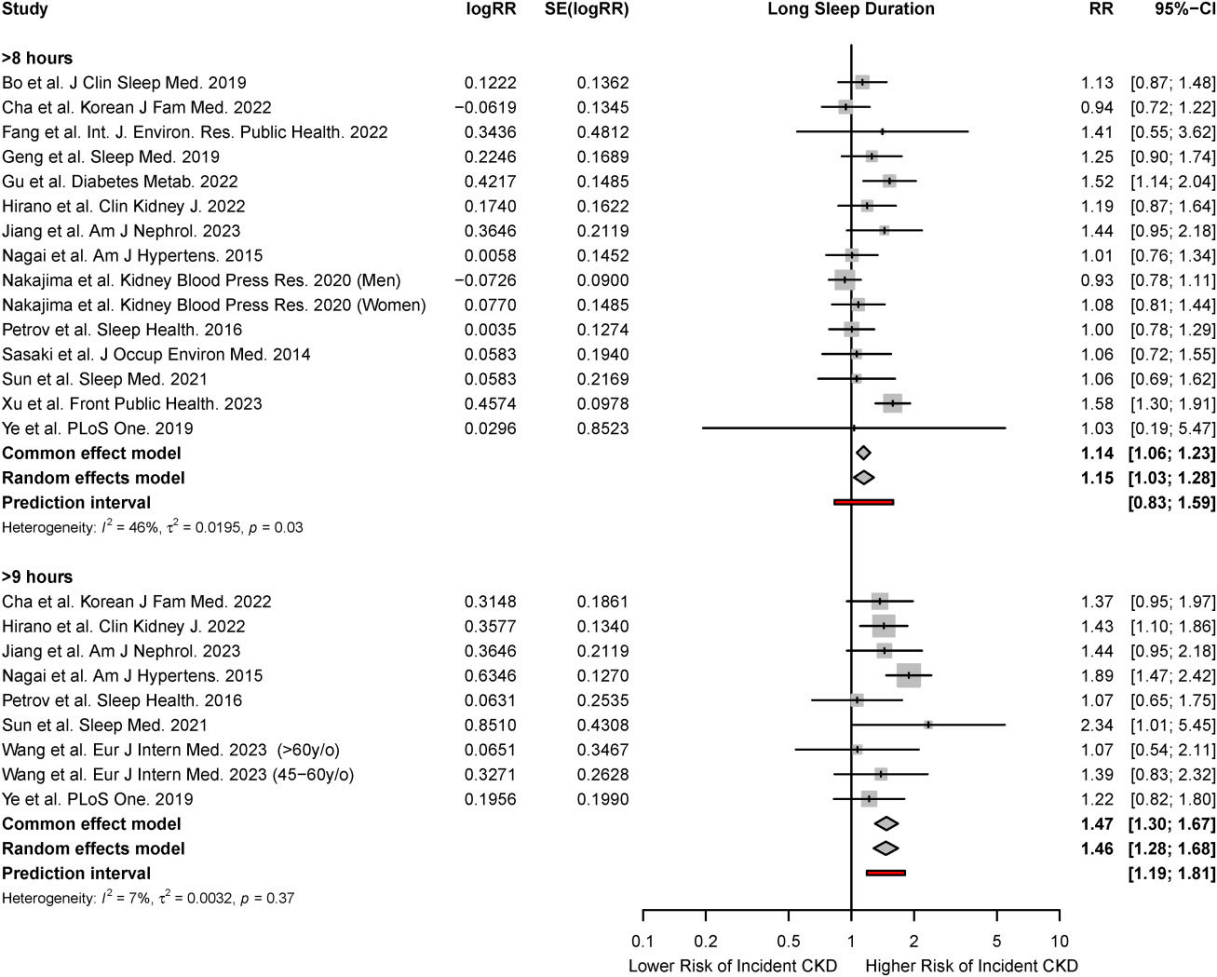
Forest plot for association of long sleep duration with risk of incident CKD.

There were 14 studies that reported the association of long sleep duration with incident CKD [9, 11, 46, 48, 49, 50, 52, 60, 61, 63, 68, 69, 76, 78]. Based on the random-effects model, long sleep durations of ≥8 hours (HR, 1.15; 95% CI, 1.03 to 1.28; *P* < 0.01, *I*^2^* = *46%) and ≥9 hours (HR, 11.46; 95% CI, 1.28 to 1.68; *P* < 0.01, *I*^2^* = *7%) were associated with a significantly increased risk of incident CKD (Fig. 3).

Meta-regression was also performed for sleep durations ≥8 hours to examine the influence of study-level covariates on the risk of incident CKD. Meta-regression found that mean age, year of study completion, percentage of male participants, percentage of obese participants, and mean BMI were not significant effect moderators of the risk of incident CKD (Supplement 4).

For sleep durations ≥8 hours, while visual inspection suggested the possibility of funnel plot asymmetry, this was not shown by Egger's test. Trim and fill resulted in minimal change to the pooled effect sizes. For all analyses, leave-one-out influence analysis showed that no single study had a drastic change on the pooled risk ratio, and cumulative meta-analysis showed a significant and stable pooled effect size (Supplements 5 and 6).

### Prevalent CKD

#### Meta-analysis for short sleep duration

**Figure 4:**
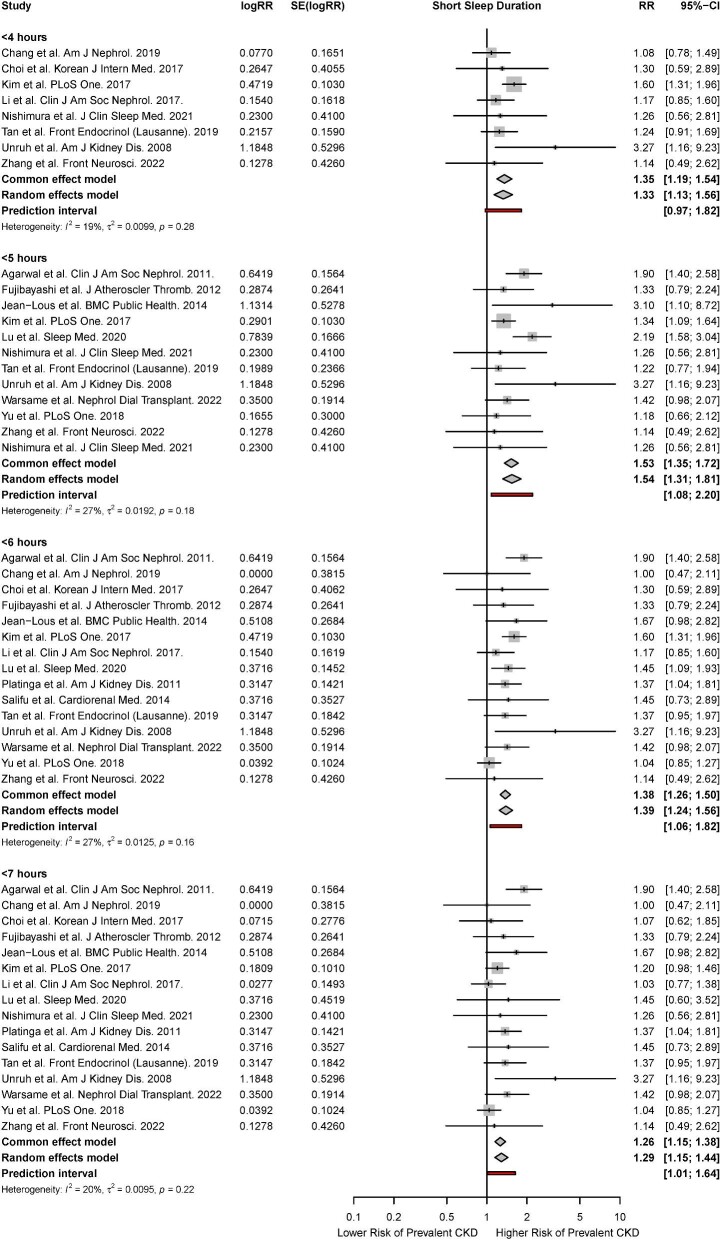
Forest plot for association of short sleep duration with risk of prevalent CKD.

There were 16 studies that reported the association of short sleep duration with the risk of prevalent CKD [10, 12, 43, 47, 51, 54, 57, 58, 62, 65, 67, 70, 72, 74, 79, 80]. Based on the random-effects model, short sleep durations of ≤4 hours (RR, 1.33; 95% CI, 1.13 to 1.56; *P* < 0.01, *I*^2^* = *19%), ≤5 hours (RR, 1.54; 95% CI, 1.31 to 1.81; *P* < 0.01, *I*^2^* = *27%), ≤6 hours (RR, 1.39; 95% CI, 1.24 to 1.56; *P* < 0.01, *I*^2^* = *27%), and ≤7 hours (RR, 1.29; 95% CI, 1.16 to 1.44; *P* < 0.01, *I*^2^* = *20%) were associated with a significantly increased risk of prevalent CKD. Significant associations were observed across both fixed- and random-effects models (Fig. 4). Meta-regression found that mean age, year of study completion, percentage of male participants, percentage of obese participants, and mean BMI were not significant effect moderators of the risk of prevalent CKD (Supplement 4). Subgroup analyses stratified by study methodology found that the association of short sleep duration with prevalent CKD remained significant across cohort and cross-sectional studies. Subgroup analyses also did not find any significant differences in association of short sleep duration with prevalent CKD between studies that used objective and subjective measures of sleep duration (Supplement 8).

#### Meta-analysis for long sleep duration

**Figure 5:**
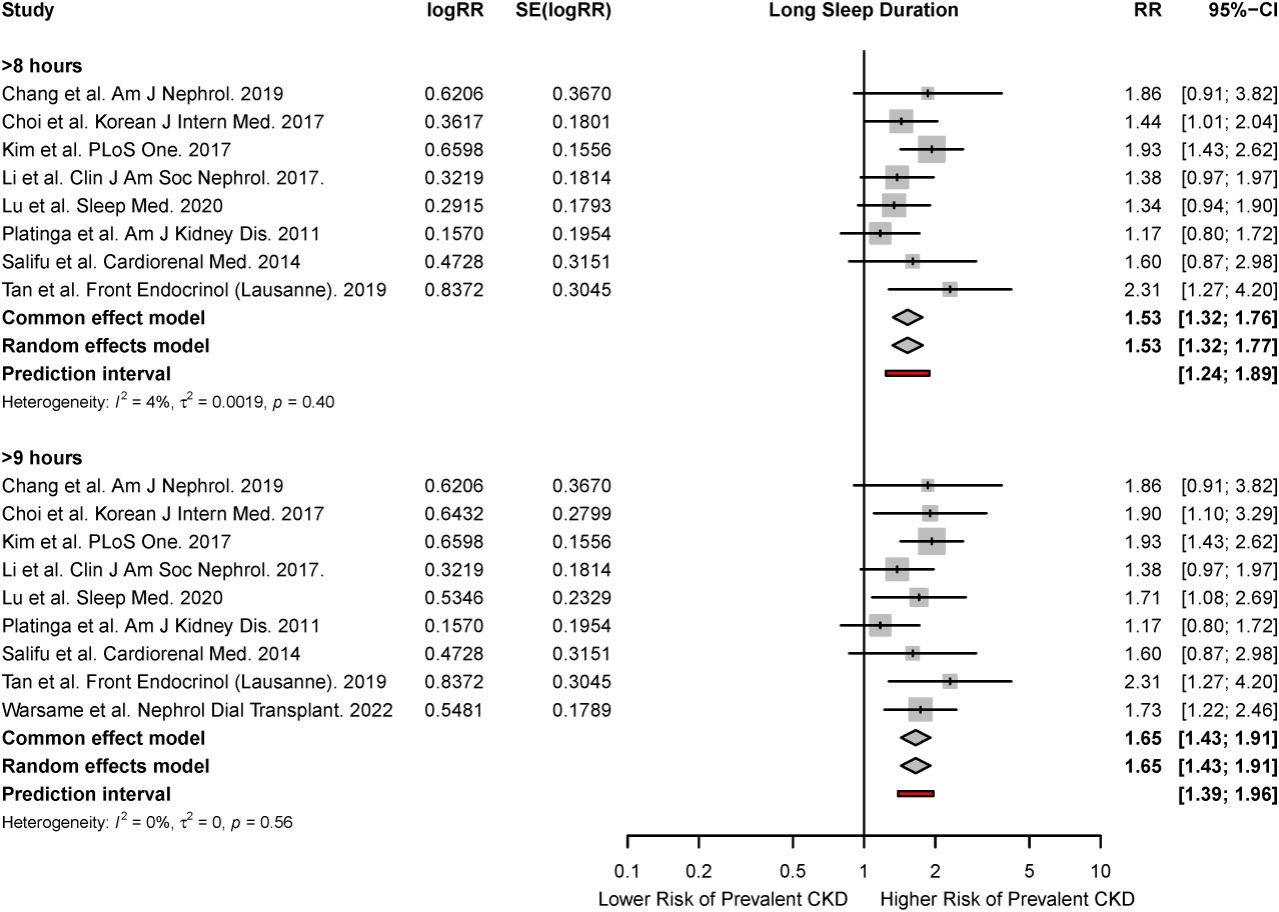
Forest plot for association of long sleep duration with risk of prevalent CKD.

There were nine studies that reported the association of long sleep duration with the risk of prevalent CKD [10, 43, 54, 57, 58, 65, 67, 70, 74]. Based on the random-effects model, long sleep durations of ≥8 hours (RR, 1.53; 95% CI, 1.32 to 1.77; *P* < 0.01, *I*^2^* = *4%) and ≥9 hours (RR, 1.65; 95% CI, 1.43 to 1.91; *P* < 0.01, *I*^2^* = *0%) were associated with a significantly increased risk of prevalent CKD. Significant associations were observed across both fixed- and random-effects models (Fig. 5).

### Meta-analysis for sleep quality

**Figure 6:**
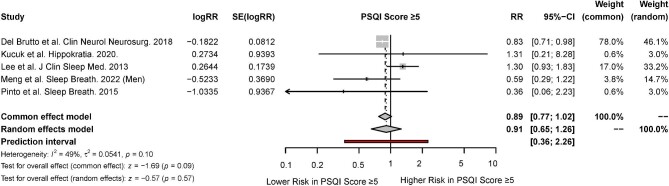
Forest plot for association of sleep quality with risk of CKD.

There were five studies that reported the association of sleep quality with CKD [45, 55, 56, 59, 64]. Based on the random-effects model, poor sleep quality as indicated by a PSQI score of ≥5 was not associated with a significantly increased risk of CKD (RR, 0.91; 95% CI, 0.65 to 1.26; *P* < 0.57, *I^P^ = *49%) (Fig. 6).

### GRADE quality of evidence

The certainty of evidence for the association of sleep duration and quality with CKD were assessed using the GRADE framework. The results of this assessment are shown in Supplement 9.

## DISCUSSION

In this systematic review and meta-analysis of 2 525 312 participants, short sleep duration and long sleep duration versus a reference range of 7 to 8 hours was associated with an increased risk of CKD. This association remained consistent across sleep durations of <4, 5, 6, and 7 hours for short sleep durations, and >8 and 9 hours for long sleep durations. Meta-regression found that the percentage of male participants and percentage of Caucasian participants were significant effect moderators of the association between short sleep duration and risk of CKD. The results of the meta-analysis were robust across different sensitivity analyses, including leave-one-out, cumulative, and trim-and-fill analyses. In 2020, Hao *et al.* investigated the relationship between sleep duration and the risk of CKD [82]. Our meta-analysis updates this review and further provides meta-analyses of the association of sleep quality with the risk of CKD. Given that insomnia and poor sleep quality often co-occur with cardiovascular diseases, psychiatric illness, and impaired social functioning that may complicate the course of CKD, identification of patients with short or excessive sleep duration may allow for early intervention to minimize delays and improve clinical outcomes and quality of life.

This meta-analysis provides robust evidence that both inadequate and excessive sleep may increase the risk of CKD in adults, with these findings being supported by previous works. Published literature has proposed several mechanisms to explain the influence of sleep deprivation on renal pathophysiology. First, the sympathetic nervous system is often overactive in situations of reduced sleep duration, which has been shown to be a risk factor for the development of CKD [83, 84]. Second, sleep deprivation predisposes to overactivity of the renin–angiotensin aldosterone system, with the resulting chronobiological alterations playing an important role in the progression of CKD [85]. Third, sleep deprivation results in elevated high-sensitivity C-reactive protein concentrations, which predisposes people to a chronic inflammatory state and increases the risk of cardiovascular illness, including CKD [86, 87]. Fourth, uremia is commonly implicated in the pathogenesis of poor sleep quality in patients with CKD. Chronic uremia is associated with neuropathy and myopathy, which predisposes people to upper airway dilator muscle dysfunction and sleep apnea [88, 89]. Peripheral neuropathy, which may arise either secondary to uremia or from the underlying cause of CKD such as diabetes, may also predispose patients to develop restless leg syndrome or periodic limb movements [90, 91]. Taken together, these findings suggest a strong pathophysiological basis for the link between accumulated sleep deficit and the risk of CKD. Nonetheless, it is plausible that among the studies that reported the prevalence of CKD, CKD itself may contribute to poor sleep [17]. Further research with larger prospective studies may be warranted to confirm the effect of poor sleep on the risk of CKD.

With regards to shift work and its influence on the risk of CKD, Sasaki *et al.* found no significant association of shift work with the risk of CKD [68]. However, short sleep duration was associated with a significantly higher risk of CKD in shift workers but not in non-shift workers, suggesting that shift work may be an effect modifier of the association between sleep duration and CKD. The basis for this association may be the disrupted circadian rhythm in shift workers, which contributes to the development of cardiovascular disease and other metabolic disorders [92, 93]. Shift work has been found to enhance the association between short sleep duration and obesity. While shift work may be associated with deleterious lifestyle factors and job strain which predisposes to the development of CKD, Sasaki *et al.* found that the risk of CKD in shift workers with short sleep duration was significantly increased even after adjustment for lifestyle factors, such as smoking status, alcohol consumption, exercise, and job strain [94, 95]. These results indicate that the interaction of shift work and short sleep duration may accelerate the development of CKD.

Interestingly, the meta-regression revealed that the percentage of male participants was a significant effect moderator of the association between short sleep duration and the risk of CKD, specifically among ≤4 hours of sleep. An increased percentage of male participants increased the risk ratio of developing CKD, among these subsets of sleep duration. Whereas published epidemiological data suggests a female preponderance for CKD, kidney function was found to decline faster in men than women. Possible reasons include unhealthier lifestyles in men, the damaging effects of testosterone, and the protective effects of estrogen on renal function [96]. Furthermore, modifiable risk factors including BMI and plasma glucose accelerate CKD progression in a greater extent in men than woman. Taken together, the interplay of these hormonal and lifestyle factors, coupled with the existing influence of short sleep duration, may result in the apparent increase in risk of CKD among male patients compared with female patients.

The meta-analysis found that long sleep duration was associated with an increased risk of CKD. This association remained significant across ≥8, ≥9, and ≥10 hours of sleep. Published literature suggests that the association between long sleep duration and CKD is multifactorial, being an interplay of physical inactivity, systemic inflammation, immune dysfunction, and disease status. For instance, long sleep duration has been associated with higher CRP and interleukin-6 levels, which are markers of a pro-inflammatory state [97]. Correspondingly, those who sleep for long periods often experience sleep fragmentation, excessive daytime sleepiness, poor sleep quality, and a sedentary lifestyle [98, 99]. The findings of this meta-analysis also support those reported by Hao *et al.* in 2020, which found a significant association between sleep >8 or 9 hours and the risk of CKD [82]. However, it should be noted that there were limited data pertaining to the association of sleep duration ≥10 hours and the risk of CKD, with only two studies being identified. Further primary studies investigating the risk of CKD in this subset of participants may be useful in confirming this apparent association between long sleep duration and the risk of CKD.

Interestingly, the meta-analysis did not find any significant association between poor sleep quality and the risk of CKD. Within the included studies, a PSQI cutoff score of 5 was selected as being indicative of poor sleep quality. The PSQI has demonstrated good sensitivity and specificity in validation studies, although it remains a subjective classification of sleep quality [100]. There is a well-established link between poor sleep quality and cardiovascular and psychiatric illness, given the mutually dependent interactions of the kidney–heart–brain crosstalk. The progression of CKD may create a vicious cycle of sleep disorders, CKD, and CKD-related complications. However, there is a lack of sufficient robust evidence for the relationship between poor sleep quality and CKD, as seen from the small number of studies identified in this systematic review. The causal relationship between sleep quality and CKD also remains debatable, as poor sleep quality may be a manifestation of major underlying factors, including a heavy burden of concomitant disease or inadequate dialysis. Within the included studies for the meta-analysis of sleep quality, there were patients who had concomitant sleep disorders, such as sleep apnea and restless leg syndrome. These sleep disorders may be effect modifiers of the relationship between sleep quality and the risk of CKD. Further well-conducted studies will be useful in elucidating this relationship between poor sleep quality and the risk of CKD.

The strengths of this study lie in the large number of systematically included studies from diverse settings. Through a rigorous prespecified protocol of systematic searching, bias assessment, and quality grading according to international guidelines, these help to enhance the generalizability of these findings. Of the 39 included studies, only seven studies were found to be of high risk of bias, and their exclusion did not change the findings to of the meta-analysis significantly. Meta-regression was able to adequately explain the observed heterogeneity in the meta-analysis, and the pooled effect sizes were robust to subgroup analyses, meta-regression, influence, and cumulative and small-study analyses. Overall, minimal evidence of publication bias was found.

There were appreciable limitations of this meta-analysis. First, the observational nature of the included studies does not permit causal conclusion due to the inability to exclude residual confounding. Second, there were unfortunately no data available for patients on transplant. Therefore, subgroup analyses comparing patients undergoing and not undergoing renal transplants could not be performed. Third, there were limited data available pertaining to the association of sleep quality with the risk of CKD, with only five studies identified in this respect. Further well-conducted studies will be useful in identifying possible associations between sleep quality and the risk of CKD. Fourth, the conduct of meta-regression was limited by the availability of published literature and could only be performed among studies that reported data on the particular covariate. Aggregate or ecological bias could not be excluded due to the assumption of linearity. Fifth, there was a lack of randomized trials identified in the literature search, which may reduce the overall strength of the meta-analysis and increase the effect of confounding factors and bias. Therefore, causal relationships cannot be inferred from this meta-analysis. The power of the meta-regression is also relatively small in the presence of confounding factors, which may increase the chance of false positive results, therefore the results of the meta-regression should be interpreted with caution.

## CONCLUSION

In this systematic review and meta-analysis of 39 studies involving 2 525 312 participants, both short and long sleep durations were significantly associated with a higher risk of CKD. This association remained significant across various sleep durations, including ≤4, ≤5, ≤6, and ≤7 hours of sleep. Similar associations were also seen for ≥8, ≥9, and ≥10 hours of sleep. Meta-regression revealed that mean age, percentage of male patients, and year of study completion did not significantly alter the pooled effect sizes. Poor sleep quality as indicated by a PSQI score of ≥5 was not associated with a higher risk of CKD. This study highlights the relationships between sleep duration and CKD and adds to the growing evidence base suggesting the interplay between both entities. Physicians treating patients with sleep disorders should be aware of these associations and adopt targeted interventions to improve clinical outcomes and quality of life in these patients.

## Supplementary Material

sfae177_Supplemental_Files

## Data Availability

All data are available from Medline/Pubmed, Embase, the Cochrane Library and CINAHL.
