# Supplementary material for: The association of sleep duration with the risk of chronic kidney disease: a systematic review and meta-analysis
Source: Clin Kidney J. 2024 Jul 11;17(8):sfae177. doi: 10.1093/ckj/sfae177 (PMC11304598; doi:10.1093/ckj/sfae177)
Supplement: sfae177_Supplemental_Files [file sfae177_supplemental_files.zip › S1. Search Strategy.docx]

**Supplement 1. Search Strategy**

**Medline (1103)**

|  | # | Search Term |
| --- | --- | --- |
| **Sleep duration** | 1 | exp sleep duration/ or (sleep and (time or duration or quantit*)).tw. |
| **Sleep quality** | 2 | exp sleep quality/ or (sleep and qualit*).tw. |
|  | 3 | 1 or 2 |
| **CKD** | 4 | exp kidney failure, chronic/ or (((kidney or renal) and (disease or failure) and (end-stage or end stage or chronic)) or ESRD).tw. |
| **Renal transplant** | 5 | exp kidney transplantation/ or (((renal or kidney) and transplant*) or (kidney and grafting)).tw. |
|  | 6 | 4 or 5 |
|  | 7 | 4 and 7 |

**Embase (2602)**

|  | # | Search Term |
| --- | --- | --- |
| **Sleep duration** | 2 | ‘sleep time’/exp OR (sleep* AND (duration OR quantity OR time)):ti,ab,kw |
| **Sleep quality** | 3 | ‘sleep quality’/exp OR (sleep* AND quality*):ti,ab,kw |
| **CKD** | 3 | 'chronic kidney failure'/exp OR (chronic:ti,ab,kw AND (renal:ti,ab,kw OR kidney:ti,ab,kw) AND (disorder:ti,ab,kw OR disease:ti,ab,kw OR insufficiency:ti,ab,kw OR failure:ti,ab,kw)) OR (chronic:ti,ab,kw AND nephropathy:ti,ab,kw) OR (kidney:ti,ab,kw AND function,:ti,ab,kw AND chronic:ti,ab,kw AND disease:ti,ab,kw) |
| **Kidney transplantation** | 4 | ‘kidney transplantation’/exp OR (kidney AND (allograft OR cadaver) AND transplantation):ti,ab,kw OR ((kidney OR renal) AND (allotransplantation OR grafting OR homotransplantation OR retransplantation OR homotransplantation OR transplantation)):ti,ab,kw OR (second set kidney transplantation):ti,ab,kw |
|  | 5 | #1 OR #2 |
|  | 6 | #3 OR #4 |
|  | 7 | #5 AND #6 |
|  | 8 | #7 AND 'article'/it |

**Cochrane (89)**

|  | # | Search Term |
| --- | --- | --- |
| Sleep duration | 2 | [mh "sleep duration"] OR (sleep:ti,ab AND (time:ti,ab OR duration:ti,ab OR quantit*:ti,ab)) |
| Sleep quality | 3 | [mh "sleep quality"] OR (sleep:ti,ab AND qualit*:ti,ab) |
|  | 4 | #1 OR #2 OR #3 |
| **CKD** | 5 | exp kidney failure, chronic/ or (((kidney or renal) and (disease or failure) and (end-stage or end stage or chronic)) or ESRD).tw. |
| **Renal transplant** | 6 | exp kidney transplantation/ or (((renal or kidney) and transplant*) or (kidney and grafting)).tw. |
|  | 7 | #5 OR #6 |
|  | 8 | #4 AND #7 |

1744 studies were identified in the initial literature search. After removal of duplicates, 1454 studies remained.
