# Supplementary material for: The association of sleep duration with the risk of chronic kidney disease: a systematic review and meta-analysis
Source: Clin Kidney J. 2024 Jul 11;17(8):sfae177. doi: 10.1093/ckj/sfae177 (PMC11304598; doi:10.1093/ckj/sfae177)
Supplement: sfae177_Supplemental_Files [file sfae177_supplemental_files.zip › S4. Results of the Meta Regression Analysis.pdf]

Supplement 4. Results of the meta-regression analysis for the association between sleep duration and risk of chronic kidney disease

Outcome	Coefficient	SE	Z	*P*	95% CI Lower	95% CI Upper	R^2 (%)	I^2 (%)
**Incident CKD**								
≤4 Hours								
Mean age	-0.0039	0.0066	-0.5936	0.5930	-0.0170	0.0091	0.00	43.72
Year of study completion	0.0056	0.0097	0.5786	0.5650	-0.0134	0.0247	0.00	67.77
Percentage male participants	2.5040	0.9709	2.5790	0.0170	0.6010	4.4070	67.22	41.35
Percentage of Caucasian participants	0.0016	0.1335	0.0117	0.9934	-0.2600	0.2631	0.00	62.12
Mean BMI	2.1311	0.6776	3.1450	0.0540	0.7581	3.5041	100.00	0.00
Percentage of obese participants	-1.5507	1.4407	-1.0764	0.2720	-4.5067	1.4053	0.00	0.00
≤5 Hours								
Mean age	-0.0231	0.0418	-0.5528	0.5190	-0.1052	0.0589	0.00	98.42
Year of study completion	-0.0026	0.0422	-0.0624	0.9180	-0.0854	0.0801	0.00	98.61
Percentage male participants	0.1318	2.457	0.0536	0.9430	-4.6872	4.9508	0.00	98.57
Percentage of Caucasian participants	0.6990	0.3634	1.9236	0.0022	-0.0132	1.4113	98.23	7.38
Mean BMI	-0.1445	1.1485	-0.1258	0.8890	-2.6724	2.3834	2.03	71.98
Percentage of obese participants	0.2783	1.3743	0.2025	0.8519	-3.5375	4.0941	64.70	0.00
≤6 Hours								
Mean age	0.0021	0.0056	0.3773	0.6910	-0.0088	0.0130	0.00	4.57
Year of study completion	0.0040	0.0129	0.3116	0.7440	-0.0213	0.0294	0.00	79.53
Percentage male participants	-0.0402	0.7971	-0.0504	0.9510	-1.6818	1.6015	60.52	0.00
Percentage of Caucasian participants	-1.2208	1.0867	-1.1234	0.3260	-3.4876	1.0460	0.00	23.26
Mean BMI	-1.7773	1.3069	-1.3600	0.1750	-4.5477	0.9931	28.09	70.89
Percentage of obese participants	-2.7522	2.6086	-1.0550	0.2970	-8.5645	3.0601	34.54	63.11
≤7 Hours								
Mean age	0.2436	0.1018	2.3936	0.0769	0.0441	0.4430	63.88	48.59
Year of study completion	0.0106	0.0079	1.3319	0.1750	-0.0050	0.0261	26.27	71.79
Percentage male participants	0.4916	1.0681	0.4603	0.6310	-1.6018	2.5850	0.00	80.54
Percentage of Caucasian participants	0.1902	0.0944	2.0158	0.0438	0.0053	0.3752	67.68	40.55
Mean BMI	0.8591	0.4562	1.8830	0.0580	-0.2198	1.9379	0.00	0.02
Percentage of obese participants	0.2913	0.4328	0.6731	0.4660	-0.6219	1.2045	54.74	0.00

≥8 Hours								
Mean age	0.0005	0.0126	0.0390	0.9780	-0.0242	0.0252	0.00	55.71
Year of study completion	-0.4380	0.1441	-3.0401	0.0700	-0.7360	-0.1400	100.00	0.00
Percentage male participants	0.2913	0.4328	0.6731	0.4660	-0.6219	1.2045	54.74	0.00
Percentage of Caucasian participants	-0.0053	0.1693	-0.0316	0.9730	-0.3625	0.3518	0.00	0.02
Mean BMI	-1.5507	1.4407	-1.0764	0.2720	-4.5067	1.4053	0.00	0.00
Percentage of obese participants	-0.1021	0.3784	-0.2699	0.7490	-0.9044	0.7001	6.10	54.35
**Prevalent CKD**								
≤4 Hours								
Mean age	0.0005	0.0048	0.1008	0.9070	-0.0097	0.0107	0.00	97.46
Year of study completion	2.1311	0.6776	3.1450	0.0540	0.7581	3.5041	100.00	0.00
Percentage male participants	-1.5507	1.4407	-1.0764	0.2720	-4.5067	1.4053	0.00	0.00
Percentage of Caucasian participants	-0.6575	0.7233	-0.9091	0.3730	-2.1416	0.8265	0.00	0.00
Mean BMI	2.1311	0.6776	3.1450	0.0540	0.7581	3.5041	100.00	0.00
Percentage of obese participants	-1.5507	1.4407	-1.0764	0.2720	-4.5067	1.4053	0.00	0.00
≤5 Hours								
Mean age	-0.0231	0.0418	-0.5528	0.5190	-0.1052	0.0589	0.00	98.42
Year of study completion	-0.0026	0.0422	-0.0624	0.9180	-0.0854	0.0801	0.00	98.61
Percentage male participants	0.1318	2.457	0.0536	0.9430	-4.6872	4.9508	0.00	98.57
Percentage of Caucasian participants	-0.3390	0.7684	-0.4412	0.6570	-1.8974	1.2194	5.13	48.71
Mean BMI	-0.1292	1.0489	-0.1232	0.9140	-2.2815	2.0230	0	45.29
Percentage of obese participants	-0.2962	0.3762	-0.7874	0.4510	-1.0680	0.4756	5.35	44.11
≤6 Hours								
Mean age	0.0021	0.0056	0.3773	0.6910	-0.0088	0.0130	0.00	4.57
Year of study completion	-0.0126	0.0101	-1.2483	0.2210	-0.0324	0.0072	11.43	44.79
Percentage male participants	-0.0254	0.0555	-0.4577	0.6810	-0.1343	0.0834	0.00	46.64
Percentage of Caucasian participants	-1.2536	0.9532	-1.3152	0.1980	-3.2743	0.7670	18.00	68.78
Mean BMI	-1.2476	1.2386	-1.0073	0.3490	-3.9463	1.4511	2.21	53.08
Percentage of obese participants	-2.7522	2.6086	-1.0550	0.2970	-8.5645	3.0601	34.54	63.11
≤7 Hours								
Mean age	0.2436	0.1018	2.3936	0.0769	0.0441	0.4430	63.88	48.59

Year of study completion	-0.0008	0.0140	-0.0540	0.9500	-0.0297	0.0282	64.44	0.00
Percentage male participants	-0.0402	0.7971	-0.0504	0.9510	-1.6818	1.6015	60.52	0.00
Percentage of Caucasian participants	-1.2208	1.0867	-1.1234	0.3260	-3.4876	1.0460	0.00	23.26
Mean BMI	0.8591	0.4562	1.8830	0.0580	-0.2198	1.9379	0.00	0.02
Percentage of obese participants	0.2913	0.4328	0.6731	0.4660	-0.6219	1.2045	54.74	0.00
