# Supplementary material for: The association of sleep duration with the risk of chronic kidney disease: a systematic review and meta-analysis
Source: Clin Kidney J. 2024 Jul 11;17(8):sfae177. doi: 10.1093/ckj/sfae177 (PMC11304598; doi:10.1093/ckj/sfae177)
Supplement: sfae177_Supplemental_Files [file sfae177_supplemental_files.zip › S5. Results of the Trim and Fill Analysis.pdf]

Supplement 5. Results of the trim and fill analysis for the association between sleep duration and risk of chronic kidney disease

Outcome	Intercept	95% CI Lower	95% CI Upper	T	*P*	N studies imputed	RR	95% CI Lower	95% CI Upper
≤4 Hours	0.0568	-0.2227	0.3363	0.33	0.7421	6	1.19	1.07	1.32
≤5 Hours	0.2466	-0.1828	0.6760	0.61	0.5580	1	1.50	1.24	1.81
≤6 Hours	0.3864	0.2145	0.5582	-0.37	0.7121	7	1.41	1.30	1.54
≤7 Hours	0.2331	-0.0771	0.3891	-0.41	0.6853	1	1.21	1.12	1.30
≥8 Hours	0.2824	-0.2197	0.34512	0.14	0.8870	1	1.36	1.23	1.51
