# Supplementary material for: The association of sleep duration with the risk of chronic kidney disease: a systematic review and meta-analysis
Source: Clin Kidney J. 2024 Jul 11;17(8):sfae177. doi: 10.1093/ckj/sfae177 (PMC11304598; doi:10.1093/ckj/sfae177)
Supplement: sfae177_Supplemental_Files [file sfae177_supplemental_files.zip › S6. Additional Analyses for Incident CKD.pdf]

Study	HR	95%−CI	P−value	Tau2	Tau	I2
Adding Bo et al. J Clin Sleep Med. 2019 (k=1)	1.52	[0.38; 6.12]	0.55	.	.	.
Adding Fang et al. Int. J. Environ. Res. Public Health. 2022 (k=2)	1.07	[0.88; 1.31]	0.50	0	0	0%
Adding Geng et al. Sleep Med. 2019 (k=3)	1.10	[0.92; 1.32]	0.27	0	0	0%
Adding Gu et al. Diabetes Metab. 2022 (k=4)	1.28	[1.00; 1.63]	0.05	0.0292	0.1710	53%
Adding Sun et al. Sleep Med. 2021 (k=5)	1.36	[1.10; 1.69]	< 0.01	0.0317	0.1782	59%
Adding Yamamoto et al. Am J Kidney Dis. 2012 (k=6)	1.41	[1.16; 1.71]	< 0.01	0.0292	0.1707	55%
**Random effects model**	**1.41**	**[1.16; 1.71]**	**< 0.01**	**0.0292**	**0.1707**	**55%**

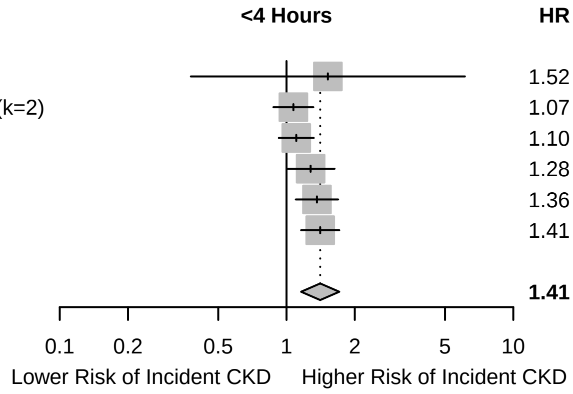

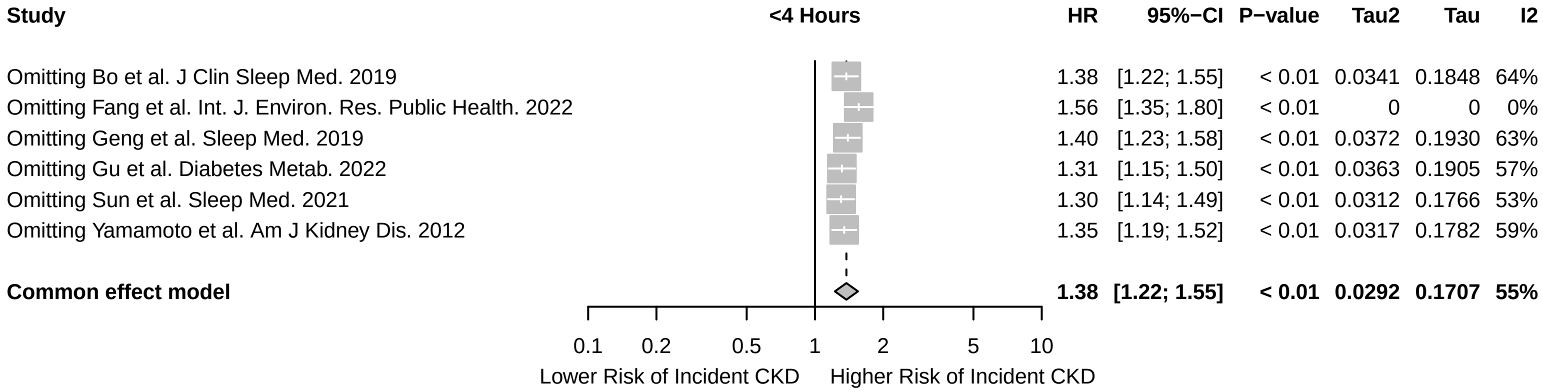
Study
<4 Hours
HR
95%−CI
P−value
Tau2
Tau
I2
Omitting Bo et al. J Clin Sleep Med. 2019 1.38 [1.22; 1.55] < 0.01 0.0341 0.1848 64%
Omitting Fang et al. Int. J. Environ. Res. Public Health. 2022 1.56 [1.35; 1.80] < 0.01 0 0 0%
Omitting Geng et al. Sleep Med. 2019 1.40 [1.23; 1.58] < 0.01 0.0372 0.1930 63%
Omitting Gu et al. Diabetes Metab. 2022 1.31 [1.15; 1.50] < 0.01 0.0363 0.1905 57%
Omitting Sun et al. Sleep Med. 2021 1.30 [1.14; 1.49] < 0.01 0.0312 0.1766 53%
Omitting Yamamoto et al. Am J Kidney Dis. 2012 1.35 [1.19; 1.52] < 0.01 0.0317 0.1782 59%
Common effect model 1.38 [1.22; 1.55] < 0.01 0.0292 0.1707 55%
0.1
0.2
0.5
1
2
5
10
Lower Risk of Incident CKD
Higher Risk of Incident CKD

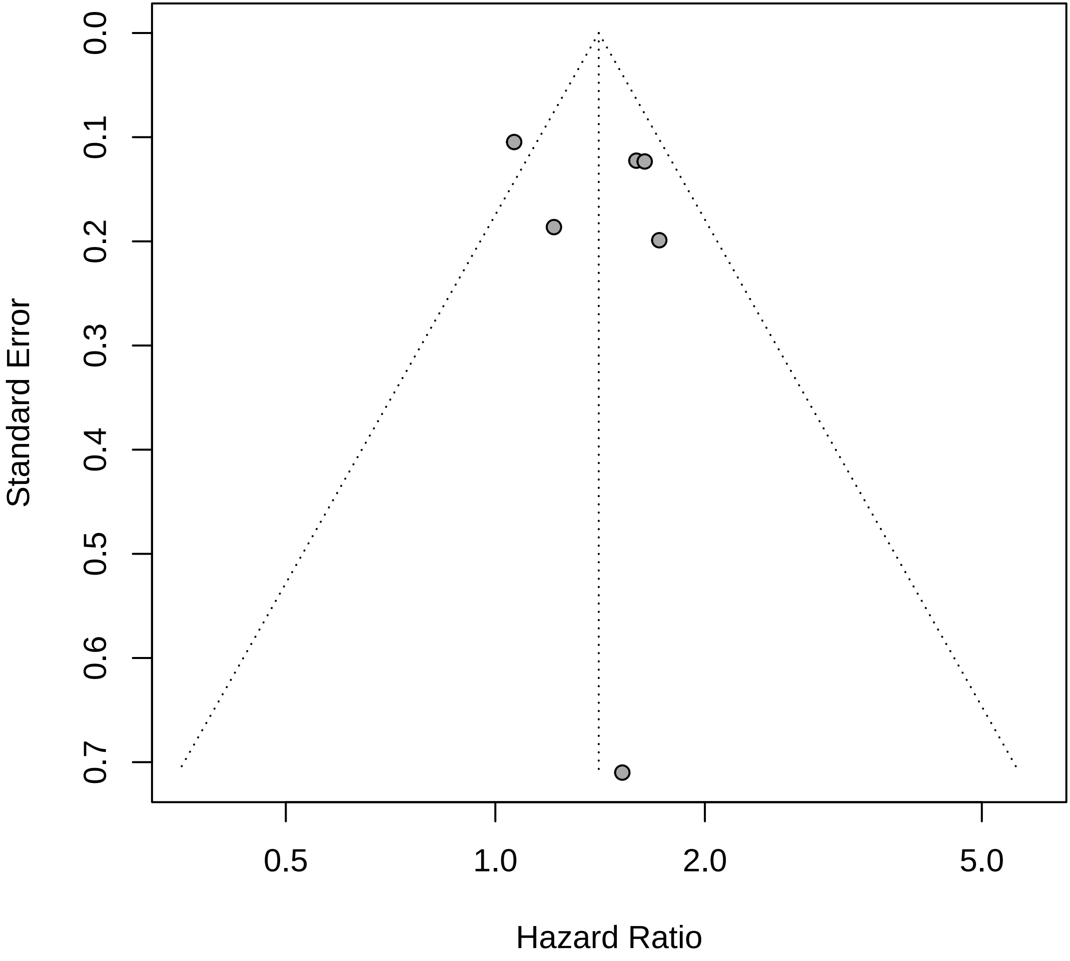

Standard Error
0.0
0.1
0.2
0.3
0.4
0.5
0.6
0.7
0.5
1.0
2.0
5.0
Hazard Ratio

Study	HR	95%−CI	P−value	Tau2	Tau	I2
Adding Bo et al. J Clin Sleep Med. 2019 (k=1)	1.52	[0.38; 6.12]	0.55	.	.	.
Adding Fang et al. Int. J. Environ. Res. Public Health. 2022 (k=2)	1.07	[0.88; 1.31]	0.50	0	0	0%
Adding Geng et al. Sleep Med. 2019 (k=3)	1.13	[0.94; 1.34]	0.19	0	0	0%
Adding Gu et al. Diabetes Metab. 2022 (k=4)	1.10	[0.95; 1.27]	0.19	0	0	0%
Adding Hirano et al. Clin Kidney J. 2022 (k=5)	1.15	[0.99; 1.34]	0.07	0.0032	0.0565	10%
Adding Petrov et al. Sleep Health. 2016 (k=6)	1.41	[1.05; 1.88]	0.02	0.0809	0.2844	72%
Adding Sun et al. Sleep Med. 2021 (k=7)	1.44	[1.12; 1.85]	< 0.01	0.0721	0.2685	73%
Adding Yamamoto et al. Am J Kidney Dis. 2012 (k=8)	1.47	[1.17; 1.85]	< 0.01	0.0669	0.2587	71%
**Random effects model**	**1.47**	**[1.17; 1.85]**	**< 0.01**	**0.0669**	**0.2587**	**71%**

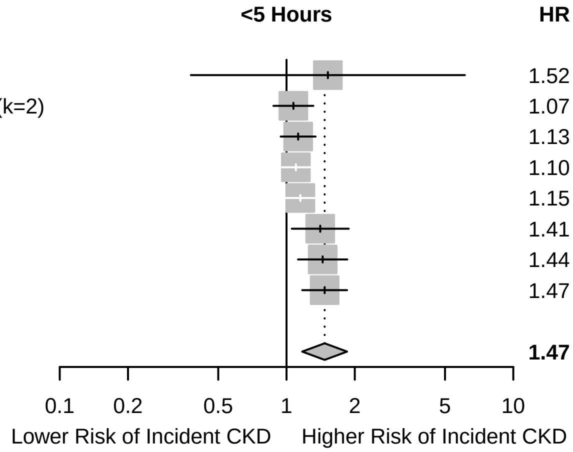

Study	<5 Hours	HR	95%−CI	P−value	Tau2	Tau	I2
Omitting Bo et al. J Clin Sleep Med. 2019		1.34	[1.20; 1.49]	< 0.01	0.0714	0.2672	75%
Omitting Fang et al. Int. J. Environ. Res. Public Health. 2022		1.47	[1.29; 1.68]	< 0.01	0.0643	0.2535	65%
Omitting Geng et al. Sleep Med. 2019		1.34	[1.19; 1.50]	< 0.01	0.0812	0.2849	75%
Omitting Gu et al. Diabetes Metab. 2022		1.43	[1.26; 1.62]	< 0.01	0.0698	0.2643	69%
Omitting Hirano et al. Clin Kidney J. 2022		1.32	[1.18; 1.48]	< 0.01	0.0716	0.2676	74%
Omitting Petrov et al. Sleep Health. 2016		1.28	[1.14; 1.43]	< 0.01	0.0319	0.1787	55%
Omitting Sun et al. Sleep Med. 2021		1.27	[1.12; 1.44]	< 0.01	0.0769	0.2774	71%
Omitting Yamamoto et al. Am J Kidney Dis. 2012		1.31	[1.17; 1.47]	< 0.01	0.0721	0.2685	73%
**Common effect model**		**1.34**	**[1.20; 1.49]**	**< 0.01**	**0.0669**	**0.2587**	**71%**

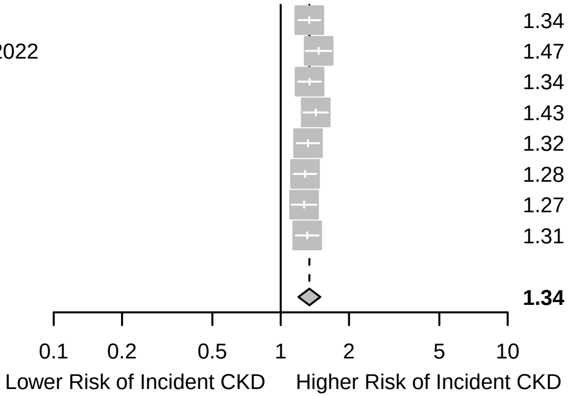

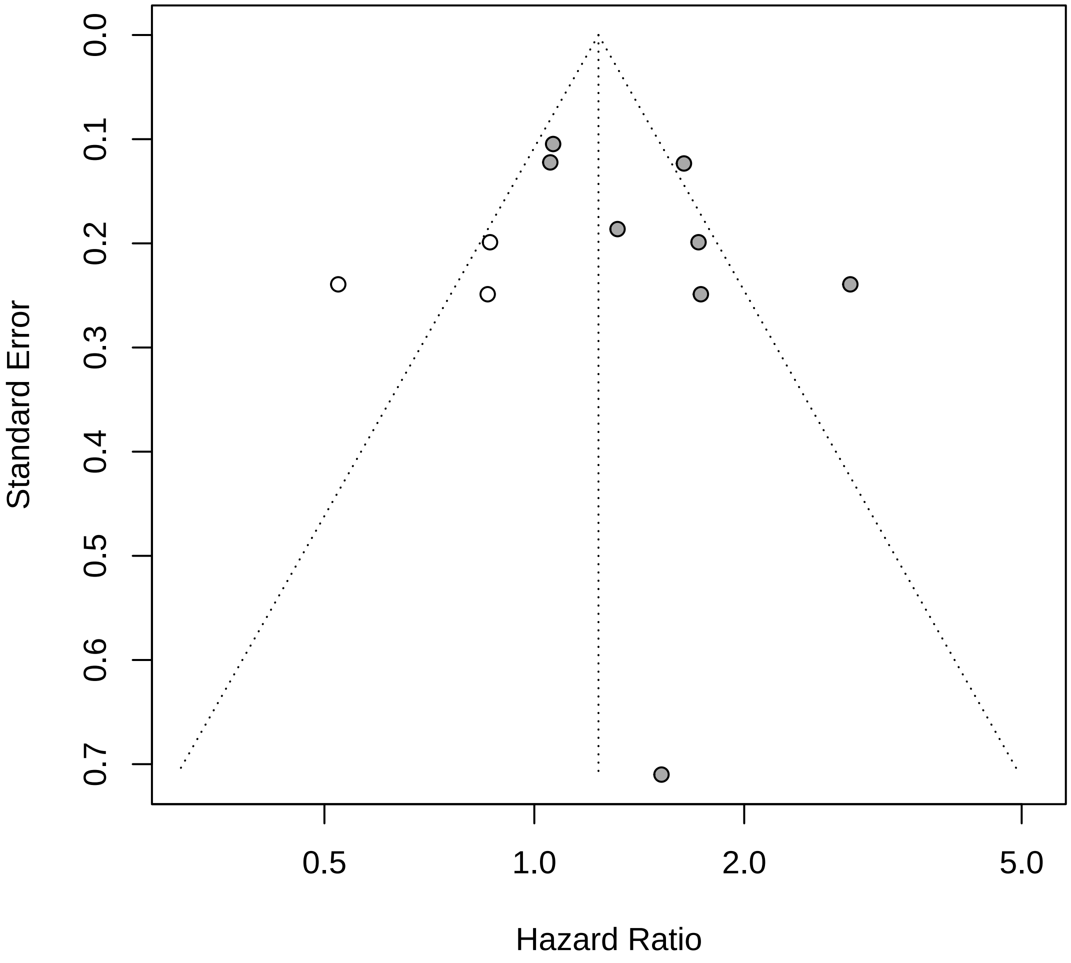

Standard Error
0.0
0.1
0.2
0.3
0.4
0.5
0.6
0.7
0.5
1.0
2.0
5.0
Hazard Ratio

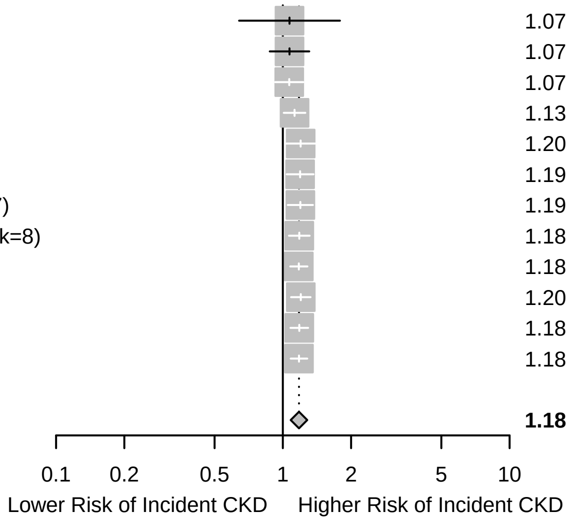

Study	<6 Hours	HR	95%−CI	P−value	Tau2	Tau	I2
Adding Bo et al. J Clin Sleep Med. 2019 (k=1)		1.07	[0.64; 1.79]	0.80	.	.	.
Adding Cha et al. Korean J Fam Med. 2022 (k=2)		1.07	[0.88; 1.31]	0.51	0	0	0%
Adding Fang et al. Int. J. Environ. Res. Public Health. 2022 (k=3)		1.07	[0.92; 1.23]	0.37	0	0	0%
Adding Geng et al. Sleep Med. 2019 (k=4)		1.13	[1.01; 1.26]	0.03	0	0	0%
Adding Gu et al. Diabetes Metab. 2022 (k=5)		1.20	[1.03; 1.39]	0.02	0.0136	0.1164	50%
Adding Hirano et al. Clin Kidney J. 2022 (k=6)		1.19	[1.04; 1.37]	0.01	0.0103	0.1015	38%
Adding Nakajima et al. Kidney Blood Press Res. 2020 (Men) (k=7)		1.19	[1.05; 1.36]	< 0.01	0.0077	0.0880	28%
Adding Nakajima et al. Kidney Blood Press Res. 2020 (Women) (k=8)		1.18	[1.06; 1.31]	< 0.01	0.0041	0.0641	18%
Adding Sun et al. Sleep Med. 2021 (k=9)		1.18	[1.08; 1.28]	< 0.01	0.0014	0.0369	7%
Adding Xu et al. Front Public Health. 2023 (k=10)		1.20	[1.09; 1.32]	< 0.01	0.0059	0.0766	25%
Adding Yamamoto et al. Am J Kidney Dis. 2012 (k=11)		1.18	[1.08; 1.29]	< 0.01	0.0048	0.0695	22%
Adding Ye et al. PLoS One. 2019 (k=12)		1.18	[1.08; 1.28]	< 0.01	0.0031	0.0560	15%
**Random effects model**		**1.18**	**[1.08; 1.28]**	**< 0.01**	**0.0031**	**0.0560**	**15%**

Study	<6 Hours	HR	95%−CI	P−value	Tau2	Tau	I2
Omitting Bo et al. J Clin Sleep Med. 2019		1.18	[1.09; 1.27]	< 0.01	0.0047	0.0686	22%
Omitting Cha et al. Korean J Fam Med. 2022		1.19	[1.10; 1.29]	< 0.01	0.0040	0.0632	17%
Omitting Fang et al. Int. J. Environ. Res. Public Health. 2022		1.19	[1.10; 1.29]	< 0.01	0.0036	0.0602	16%
Omitting Geng et al. Sleep Med. 2019		1.17	[1.07; 1.27]	< 0.01	0.0057	0.0753	21%
Omitting Gu et al. Diabetes Metab. 2022		1.14	[1.05; 1.23]	< 0.01	0	0	0%
Omitting Hirano et al. Clin Kidney J. 2022		1.18	[1.09; 1.27]	< 0.01	0.0047	0.0683	22%
Omitting Nakajima et al. Kidney Blood Press Res. 2020 (Men)		1.17	[1.09; 1.27]	< 0.01	0.0044	0.0662	21%
Omitting Nakajima et al. Kidney Blood Press Res. 2020 (Women)		1.18	[1.09; 1.28]	< 0.01	0.0052	0.0721	21%
Omitting Sun et al. Sleep Med. 2021		1.18	[1.09; 1.28]	< 0.01	0.0055	0.0743	22%
Omitting Xu et al. Front Public Health. 2023		1.16	[1.08; 1.25]	< 0.01	0	0	0%
Omitting Yamamoto et al. Am J Kidney Dis. 2012		1.19	[1.10; 1.29]	< 0.01	0.0039	0.0627	17%
Omitting Ye et al. PLoS One. 2019		1.18	[1.09; 1.27]	< 0.01	0.0048	0.0695	22%
**Common effect model**		**1.18**	**[1.09; 1.27]**	**< 0.01**	**0.0031**	**0.0560**	**15%**

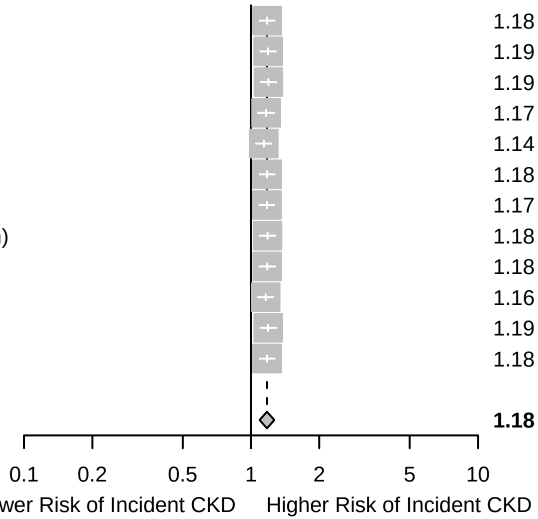

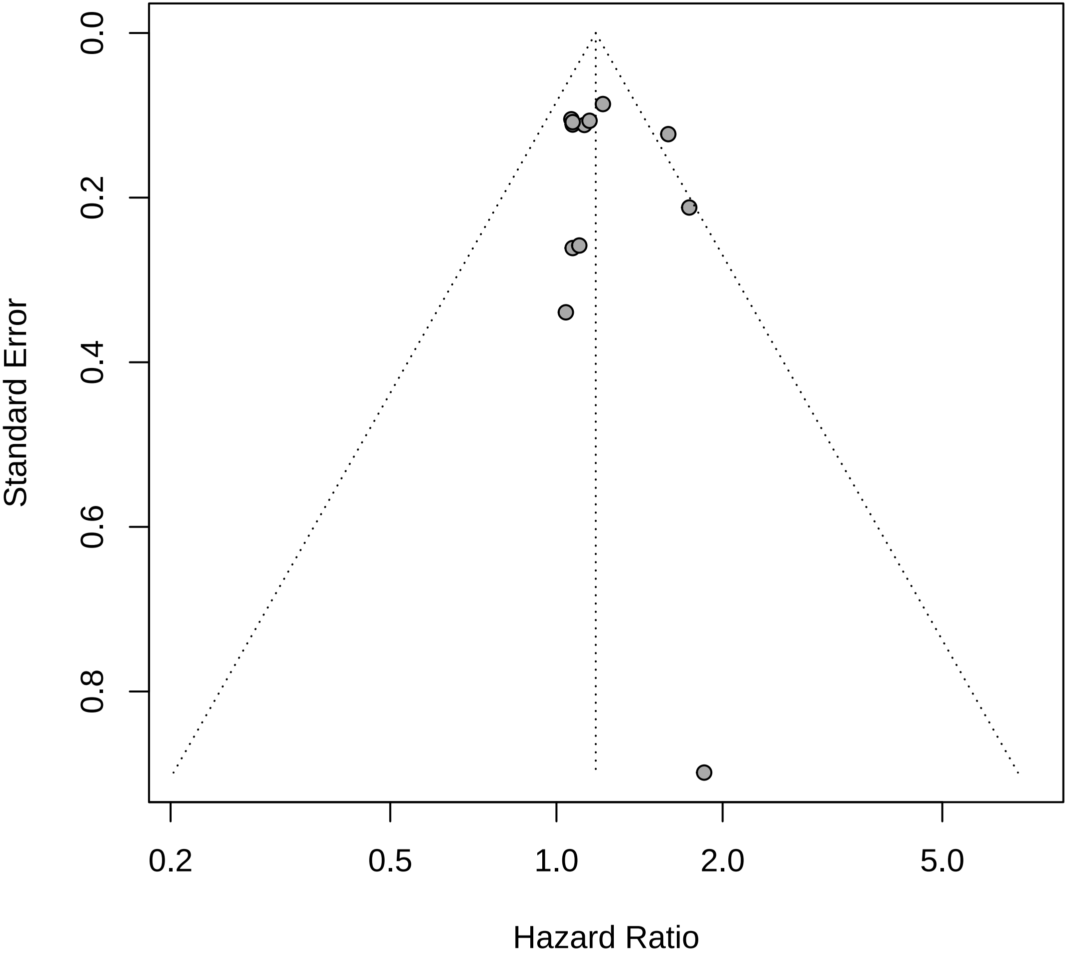

0.0
0.2
0.4
0.6
0.8
Standard Error
0.2
0.5
1.0
2.0
5.0
Hazard Ratio

Study	HR	95%−CI	P−value	Tau2	Tau	I2
Adding Bo et al. J Clin Sleep Med. 2019 (k=1)	1.07	[0.64; 1.79]	0.80	.	.	.
Adding Cha et al. Korean J Fam Med. 2022 (k=2)	1.07	[0.88; 1.31]	0.51	0	0	0%
Adding Fang et al. Int. J. Environ. Res. Public Health. 2022 (k=3)	1.07	[0.92; 1.23]	0.37	0	0	0%
Adding Geng et al. Sleep Med. 2019 (k=4)	1.13	[1.01; 1.25]	0.03	0	0	0%
Adding Gu et al. Diabetes Metab. 2022 (k=5)	1.14	[1.04; 1.26]	< 0.01	0	0	0%
Adding Hirano et al. Clin Kidney J. 2022 (k=6)	1.16	[1.06; 1.28]	< 0.01	0	0	0%
Adding Jiang et al. Am J Nephrol. 2023 (k=7)	1.22	[1.09; 1.35]	< 0.01	0.0063	0.0791	32%
Adding Nagai et al. Am J Hypertens. 2015 (k=8)	1.19	[1.08; 1.32]	< 0.01	0.0071	0.0845	35%
Adding Nakajima et al. Kidney Blood Press Res. 2020 (Men) (k=9)	1.19	[1.08; 1.32]	< 0.01	0.0057	0.0752	27%
Adding Nakajima et al. Kidney Blood Press Res. 2020 (Women) (k=10)	1.19	[1.09; 1.29]	< 0.01	0.0037	0.0607	20%
Adding Petrov et al. Sleep Health. 2016 (k=11)	1.19	[1.10; 1.30]	< 0.01	0.0033	0.0578	17%
Adding Sun et al. Sleep Med. 2021 (k=12)	1.19	[1.10; 1.28]	< 0.01	0.0017	0.0408	10%
Adding Xu et al. Front Public Health. 2023 (k=13)	1.20	[1.11; 1.31]	< 0.01	0.0045	0.0674	22%
Adding Yamamoto et al. Am J Kidney Dis. 2012 (k=14)	1.19	[1.10; 1.28]	< 0.01	0.0041	0.0641	20%
Adding Ye et al. PLoS One. 2019 (k=15)	1.19	[1.10; 1.28]	< 0.01	0.0029	0.0541	15%
**Random effects model**	**1.19**	**[1.10; 1.28]**	**< 0.01**	**0.0029**	**0.0541**	**15%**

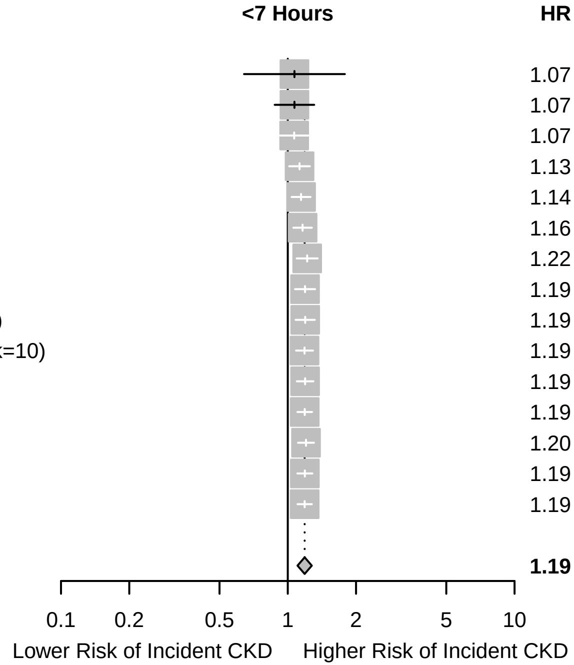

Study	<7 Hours	HR	95%−CI	P−value	Tau2	Tau	I2
Omitting Bo et al. J Clin Sleep Med. 2019		1.19	[1.11; 1.27]	< 0.01	0.0040	0.0634	20%
Omitting Cha et al. Korean J Fam Med. 2022		1.20	[1.12; 1.28]	< 0.01	0.0033	0.0576	16%
Omitting Fang et al. Int. J. Environ. Res. Public Health. 2022		1.20	[1.12; 1.29]	< 0.01	0.0030	0.0549	15%
Omitting Geng et al. Sleep Med. 2019		1.18	[1.10; 1.27]	< 0.01	0.0050	0.0706	21%
Omitting Gu et al. Diabetes Metab. 2022		1.18	[1.11; 1.26]	< 0.01	0.0044	0.0661	20%
Omitting Hirano et al. Clin Kidney J. 2022		1.18	[1.10; 1.26]	< 0.01	0.0013	0.0364	8%
Omitting Jiang et al. Am J Nephrol. 2023		1.15	[1.07; 1.23]	< 0.01	0	0	0%
Omitting Nagai et al. Am J Hypertens. 2015		1.20	[1.12; 1.28]	< 0.01	0.0023	0.0484	12%
Omitting Nakajima et al. Kidney Blood Press Res. 2020 (Men)		1.19	[1.11; 1.27]	< 0.01	0.0038	0.0620	20%
Omitting Nakajima et al. Kidney Blood Press Res. 2020 (Women)		1.19	[1.12; 1.28]	< 0.01	0.0042	0.0651	20%
Omitting Petrov et al. Sleep Health. 2016		1.18	[1.11; 1.26]	< 0.01	0.0031	0.0560	16%
Omitting Sun et al. Sleep Med. 2021		1.19	[1.11; 1.27]	< 0.01	0.0045	0.0670	20%
Omitting Xu et al. Front Public Health. 2023		1.18	[1.10; 1.25]	< 0.01	0.0001	0.0109	1%
Omitting Yamamoto et al. Am J Kidney Dis. 2012		1.20	[1.12; 1.28]	< 0.01	0.0033	0.0571	16%
Omitting Ye et al. PLoS One. 2019		1.19	[1.11; 1.27]	< 0.01	0.0041	0.0641	20%
**Common effect model**		**1.19**	**[1.11; 1.27]**	**< 0.01**	**0.0029**	**0.0541**	**15%**

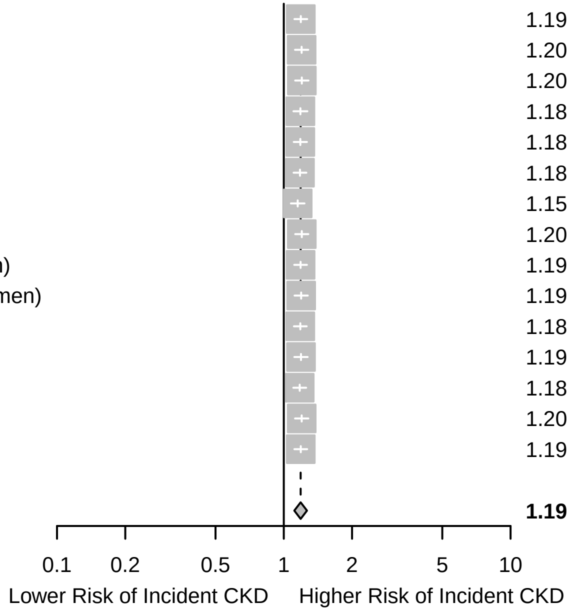

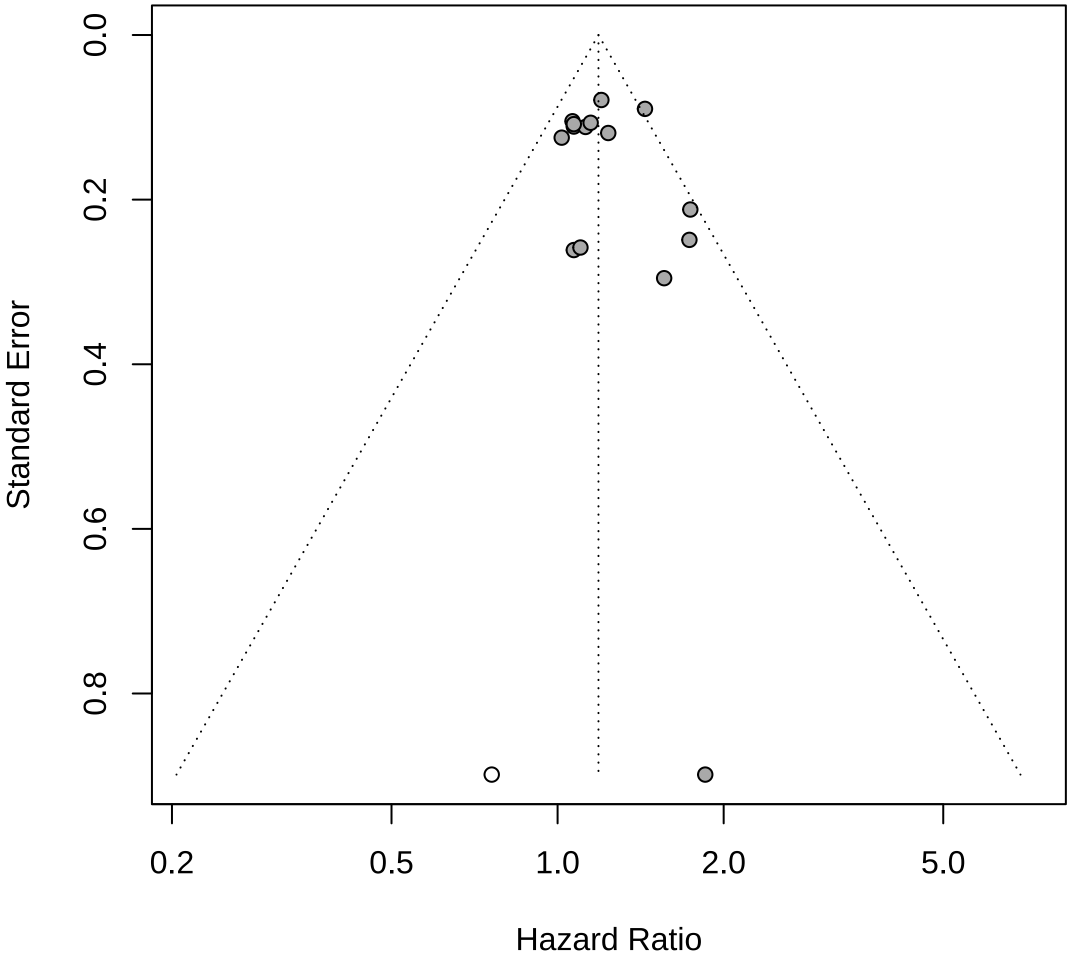

0.0
0.2
0.4
0.6
0.8
Standard Error
0.2
0.5
1.0
2.0
5.0
Hazard Ratio

Study	HR	95%−CI	P−value	Tau2	Tau	I2
Adding Fang et al. Int. J. Environ. Res. Public Health. 2022 (k=1)	1.41	[0.55; 3.62]	0.48	.	.	.
Adding Geng et al. Sleep Med. 2019 (k=2)	1.27	[0.93; 1.73]	0.14	0	0	0%
Adding Gu et al. Diabetes Metab. 2022 (k=3)	1.40	[1.13; 1.73]	< 0.01	0	0	0%
Adding Hirano et al. Clin Kidney J. 2022 (k=4)	1.24	[1.11; 1.38]	< 0.01	0	0	0%
Adding Jiang et al. Am J Nephrol. 2023 (k=5)	1.25	[1.13; 1.39]	< 0.01	0	0	0%
Adding Nagai et al. Am J Hypertens. 2015 (k=6)	1.22	[1.11; 1.34]	< 0.01	0	0	0%
Adding Petrov et al. Sleep Health. 2016 (k=7)	1.19	[1.07; 1.33]	< 0.01	0.0030	0.0547	14%
Adding Sun et al. Sleep Med. 2021 (k=8)	1.18	[1.08; 1.30]	< 0.01	0.0006	0.0245	3%
Adding Xu et al. Front Public Health. 2023 (k=9)	1.25	[1.10; 1.41]	< 0.01	0.0142	0.1191	45%
Adding Ye et al. PLoS One. 2019 (k=10)	1.24	[1.10; 1.40]	< 0.01	0.0122	0.1104	38%
**Random effects model**	**1.24**	**[1.10; 1.40]**	**< 0.01**	**0.0122**	**0.1104**	**38%**

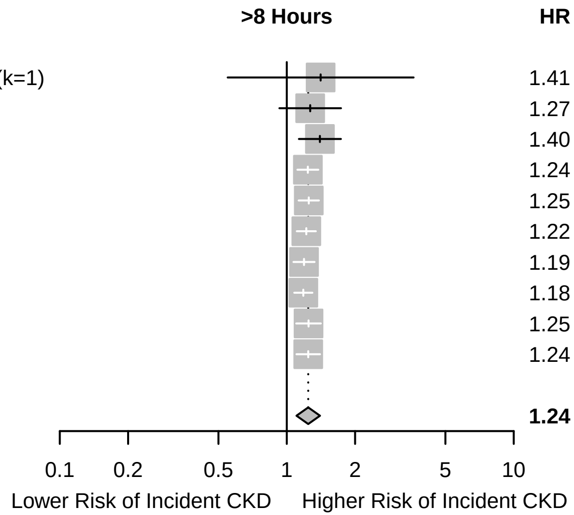

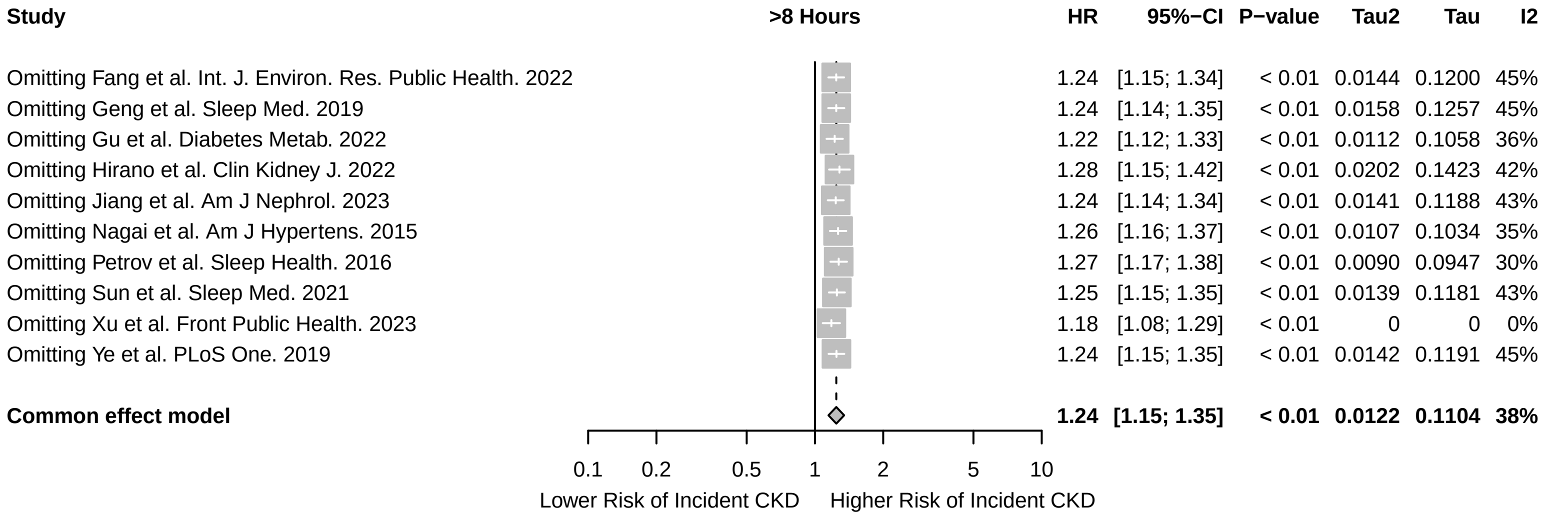
Study
>8 Hours
HR
95%−CI
P−value
Tau2
Tau
I2
Omitting Fang et al. Int. J. Environ. Res. Public Health. 2022 1.24 [1.15; 1.34] < 0.01 0.0144 0.1200 45%
Omitting Geng et al. Sleep Med. 2019 1.24 [1.14; 1.35] < 0.01 0.0158 0.1257 45%
Omitting Gu et al. Diabetes Metab. 2022 1.22 [1.12; 1.33] < 0.01 0.0112 0.1058 36%
Omitting Hirano et al. Clin Kidney J. 2022 1.28 [1.15; 1.42] < 0.01 0.0202 0.1423 42%
Omitting Jiang et al. Am J Nephrol. 2023 1.24 [1.14; 1.34] < 0.01 0.0141 0.1188 43%
Omitting Nagai et al. Am J Hypertens. 2015 1.26 [1.16; 1.37] < 0.01 0.0107 0.1034 35%
Omitting Petrov et al. Sleep Health. 2016 1.27 [1.17; 1.38] < 0.01 0.0090 0.0947 30%
Omitting Sun et al. Sleep Med. 2021 1.25 [1.15; 1.35] < 0.01 0.0139 0.1181 43%
Omitting Xu et al. Front Public Health. 2023 1.18 [1.08; 1.29] < 0.01 0 0 0%
Omitting Ye et al. PLoS One. 2019 1.24 [1.15; 1.35] < 0.01 0.0142 0.1191 45%
Common effect model 1.24 [1.15; 1.35] < 0.01 0.0122 0.1104 38%
0.1 0.2 0.5 1 2 5 10
Lower Risk of Incident CKD
Higher Risk of Incident CKD

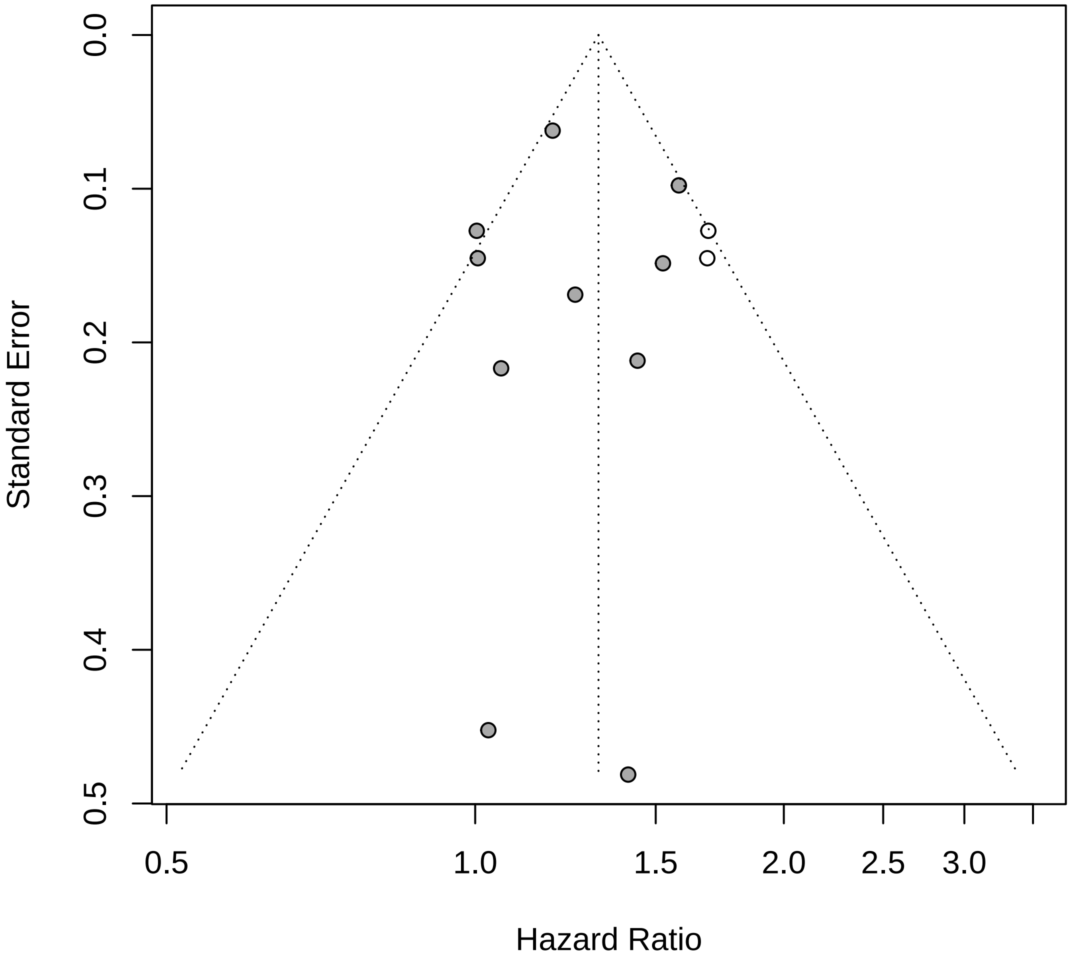

Standard Error
0.0
0.1
0.2
0.3
0.4
0.5
0.5
1.0
1.5
2.0
2.5
3.0
Hazard Ratio

Study	HR	95%−CI	P−value	Tau2	Tau	I2
Adding Hirano et al. Clin Kidney J. 2022 (k=1)	1.43	[1.10; 1.86]	< 0.01	.	.	.
Adding Jiang et al. Am J Nephrol. 2023 (k=2)	1.43	[1.15; 1.79]	< 0.01	0	0	0%
Adding Nagai et al. Am J Hypertens. 2015 (k=3)	1.61	[1.33; 1.95]	< 0.01	0.0070	0.0839	23%
Adding Petrov et al. Sleep Health. 2016 (k=4)	1.51	[1.22; 1.87]	< 0.01	0.0194	0.1394	41%
Adding Sun et al. Sleep Med. 2021 (k=5)	1.55	[1.26; 1.90]	< 0.01	0.0172	0.1312	33%
Adding Ye et al. PLoS One. 2019 (k=6)	1.49	[1.23; 1.79]	< 0.01	0.0169	0.1302	32%
**Random effects model**	**1.49**	**[1.23; 1.79]**	**< 0.01**	**0.0169**	**0.1302**	**32%**

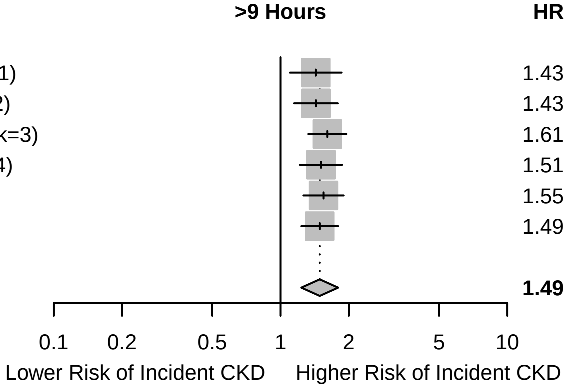

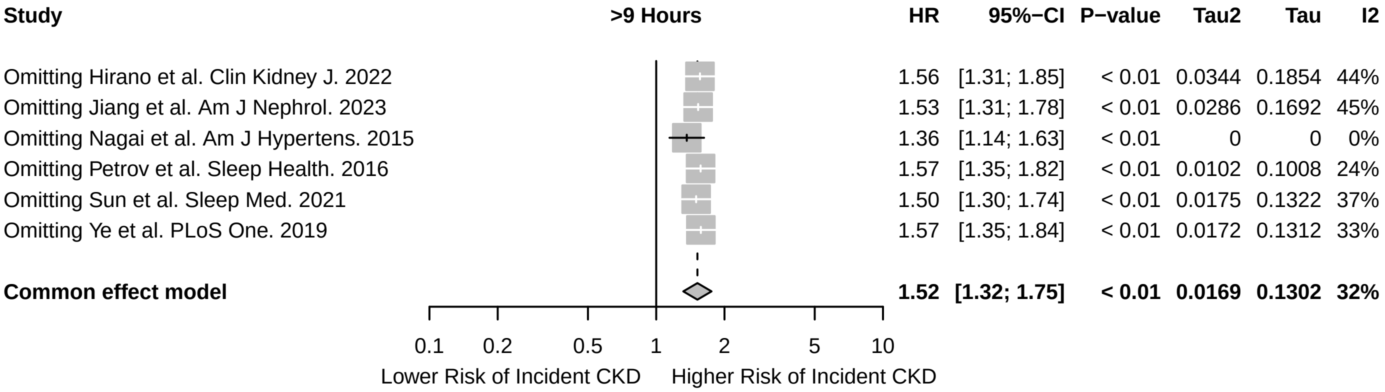
Study
>9 Hours
HR
95%−CI
P−value
Tau2
Tau
I2
Omitting Hirano et al. Clin Kidney J. 2022 1.56 [1.31; 1.85] < 0.01 0.0344 0.1854 44%
Omitting Jiang et al. Am J Nephrol. 2023 1.53 [1.31; 1.78] < 0.01 0.0286 0.1692 45%
Omitting Nagai et al. Am J Hypertens. 2015 1.36 [1.14; 1.63] < 0.01 0 0 0%
Omitting Petrov et al. Sleep Health. 2016 1.57 [1.35; 1.82] < 0.01 0.0102 0.1008 24%
Omitting Sun et al. Sleep Med. 2021 1.50 [1.30; 1.74] < 0.01 0.0175 0.1322 37%
Omitting Ye et al. PLoS One. 2019 1.57 [1.35; 1.84] < 0.01 0.0172 0.1312 33%
Common effect model 1.52 [1.32; 1.75] < 0.01 0.0169 0.1302 32%
0.1
0.2
0.5
1
2
5
10
Lower Risk of Incident CKD
Higher Risk of Incident CKD

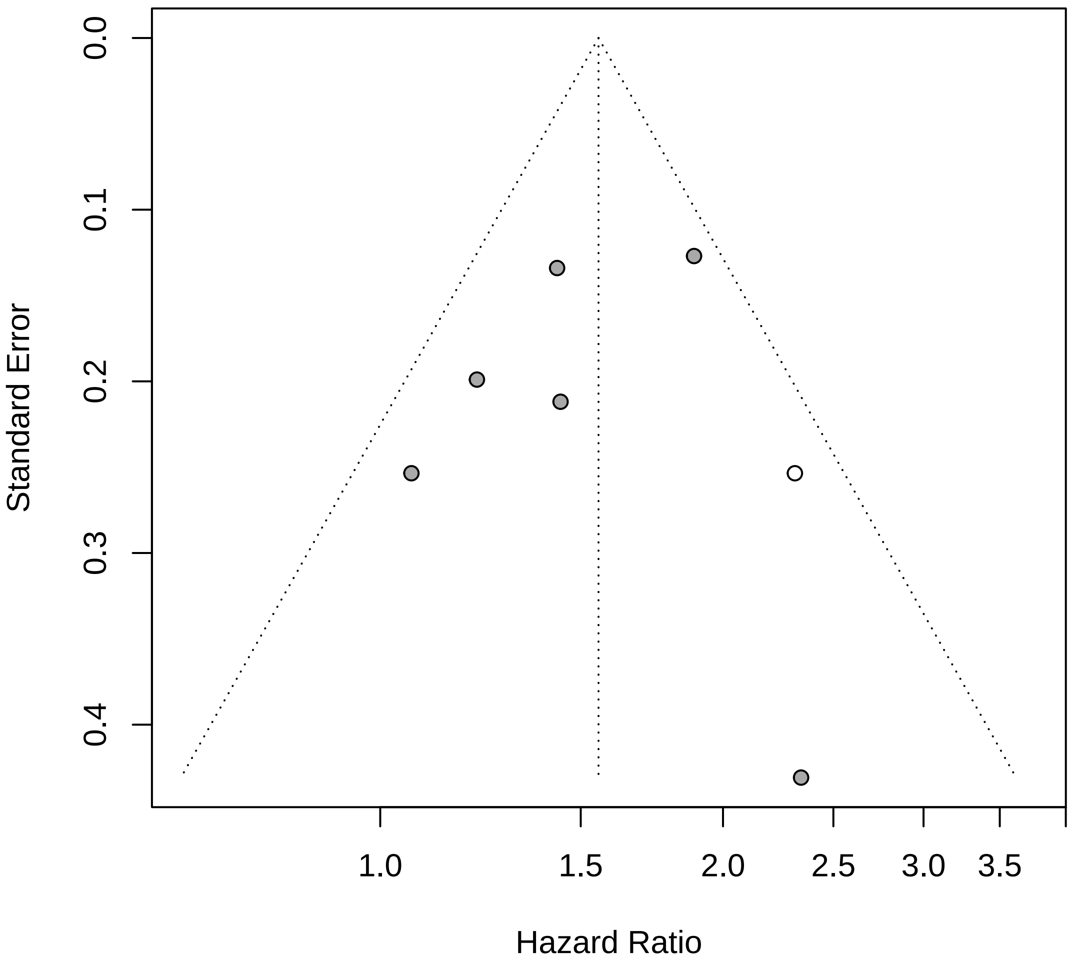

Standard Error
0.0
0.1
0.2
0.3
0.4
1.0
1.5
2.0
2.5
3.0
3.5
Hazard Ratio
