# Supplementary material for: The association of sleep duration with the risk of chronic kidney disease: a systematic review and meta-analysis
Source: Clin Kidney J. 2024 Jul 11;17(8):sfae177. doi: 10.1093/ckj/sfae177 (PMC11304598; doi:10.1093/ckj/sfae177)
Supplement: sfae177_Supplemental_Files [file sfae177_supplemental_files.zip › S7. Additional Analyses for Prevalent CKD.pdf]

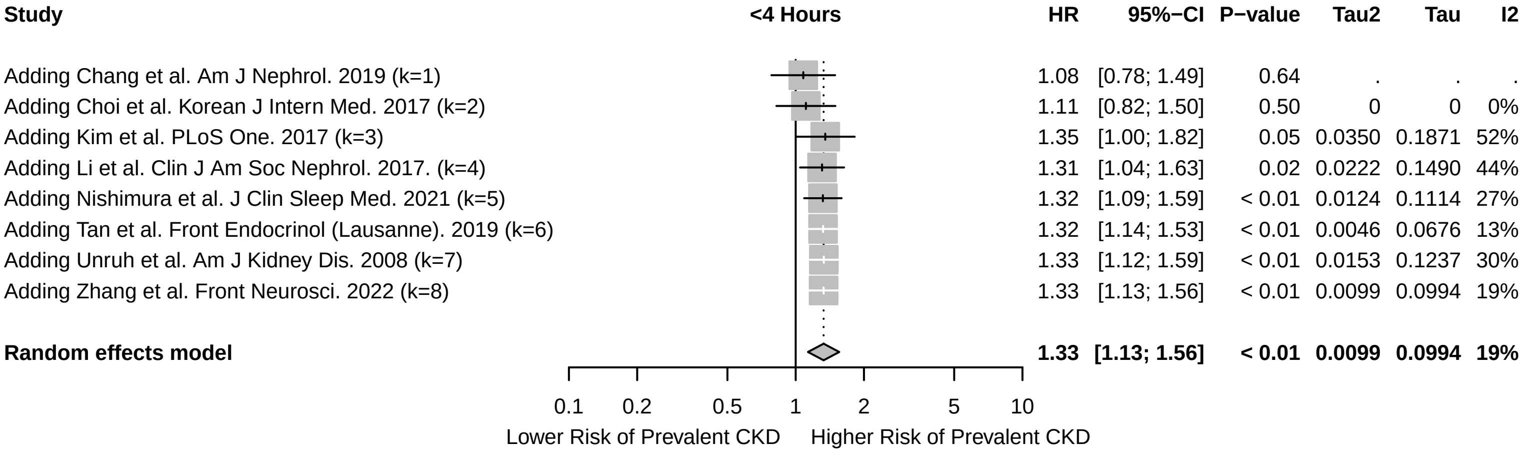
Study
<4 Hours
HR
95%−CI
P−value
Tau2
Tau
I2
Adding Chang et al. Am J Nephrol. 2019 (k=1) 1.08 [0.78; 1.49] 0.64 . . .
Adding Choi et al. Korean J Intern Med. 2017 (k=2) 1.11 [0.82; 1.50] 0.50 0 0 0%
Adding Kim et al. PLoS One. 2017 (k=3) 1.35 [1.00; 1.82] 0.05 0.0350 0.1871 52%
Adding Li et al. Clin J Am Soc Nephrol. 2017. (k=4) 1.31 [1.04; 1.63] 0.02 0.0222 0.1490 44%
Adding Nishimura et al. J Clin Sleep Med. 2021 (k=5) 1.32 [1.09; 1.59] < 0.01 0.0124 0.1114 27%
Adding Tan et al. Front Endocrinol (Lausanne). 2019 (k=6) 1.32 [1.14; 1.53] < 0.01 0.0046 0.0676 13%
Adding Unruh et al. Am J Kidney Dis. 2008 (k=7) 1.33 [1.12; 1.59] < 0.01 0.0153 0.1237 30%
Adding Zhang et al. Front Neurosci. 2022 (k=8) 1.33 [1.13; 1.56] < 0.01 0.0099 0.0994 19%
Random effects model 1.33 [1.13; 1.56] < 0.01 0.0099 0.0994 19%
0.1
0.2
0.5
1
2
5
10
Lower Risk of Prevalent CKD
Higher Risk of Prevalent CKD

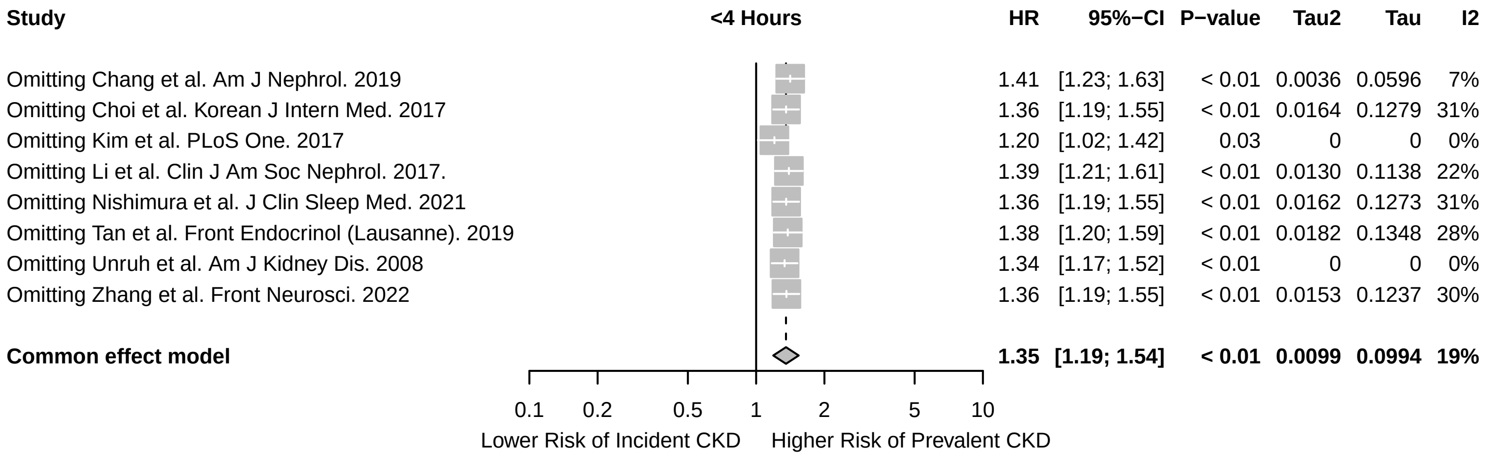
Study
<4 Hours
HR
95%−CI
P−value
Tau2
Tau
I2
Omitting Chang et al. Am J Nephrol. 2019 1.41 [1.23; 1.63] < 0.01 0.0036 0.0596 7%
Omitting Choi et al. Korean J Intern Med. 2017 1.36 [1.19; 1.55] < 0.01 0.0164 0.1279 31%
Omitting Kim et al. PLoS One. 2017 1.20 [1.02; 1.42] 0.03 0 0 0%
Omitting Li et al. Clin J Am Soc Nephrol. 2017. 1.39 [1.21; 1.61] < 0.01 0.0130 0.1138 22%
Omitting Nishimura et al. J Clin Sleep Med. 2021 1.36 [1.19; 1.55] < 0.01 0.0162 0.1273 31%
Omitting Tan et al. Front Endocrinol (Lausanne). 2019 1.38 [1.20; 1.59] < 0.01 0.0182 0.1348 28%
Omitting Unruh et al. Am J Kidney Dis. 2008 1.34 [1.17; 1.52] < 0.01 0 0 0%
Omitting Zhang et al. Front Neurosci. 2022 1.36 [1.19; 1.55] < 0.01 0.0153 0.1237 30%
Common effect model 1.35 [1.19; 1.54] < 0.01 0.0099 0.0994 19%
0.1
0.2
0.5
1
2
5
10
Lower Risk of Incident CKD
Higher Risk of Prevalent CKD

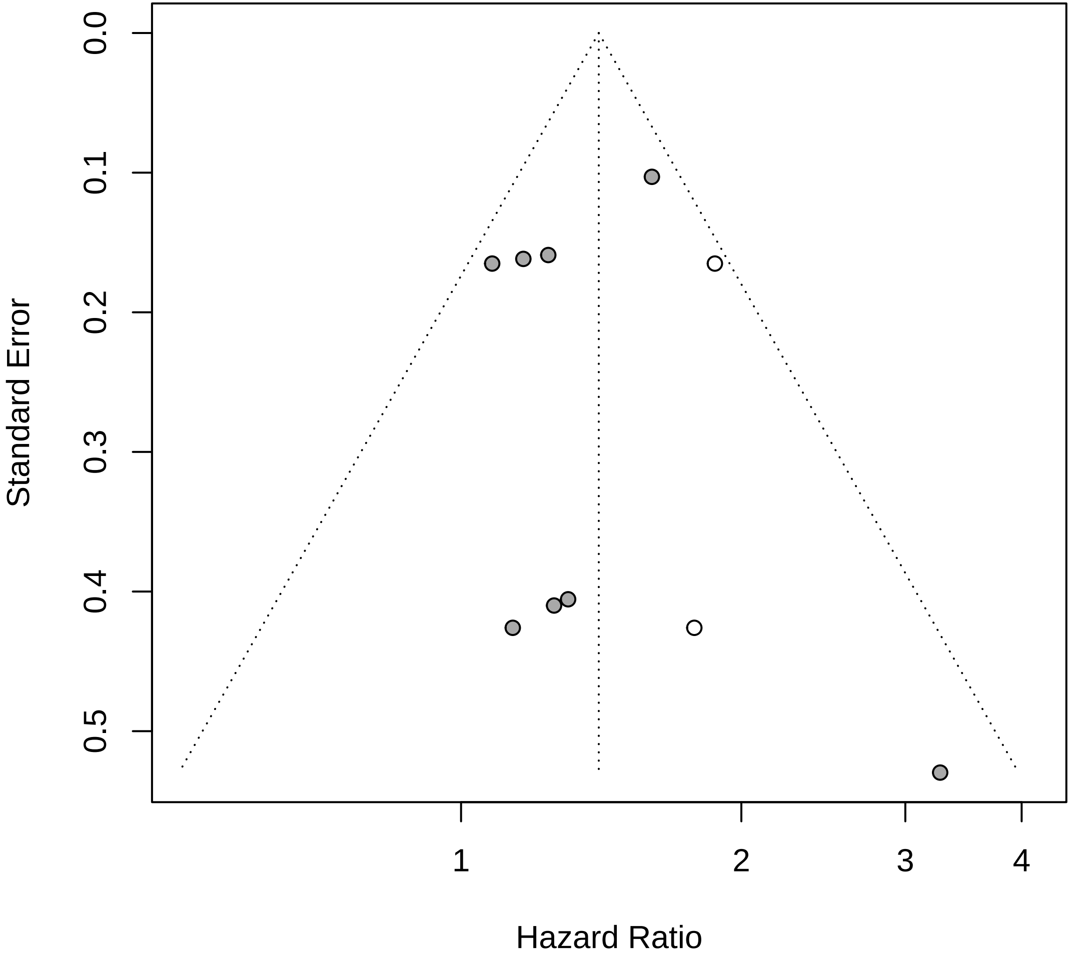

0.0
0.1
0.2
0.3
0.4
0.5
Standard Error
1
2
3
4
Hazard Ratio

Study	HR	95%−CI	P−value	Tau2	Tau	I2
Adding Agarwal et al. Clin J Am Soc Nephrol. 2011. (k=1)	1.90	[1.40; 2.58]	< 0.01	.	.	.
Adding Fujibayashi et al. J Atheroscler Thromb. 2012 (k=2)	1.70	[1.23; 2.35]	< 0.01	0.0157	0.1254	25%
Adding Jean−Lous et al. BMC Public Health. 2014 (k=3)	1.79	[1.30; 2.45]	< 0.01	0.0178	0.1333	19%
Adding Kim et al. PLoS One. 2017 (k=4)	1.58	[1.21; 2.05]	< 0.01	0.0318	0.1784	47%
Adding Lu et al. Sleep Med. 2020 (k=5)	1.72	[1.32; 2.23]	< 0.01	0.0467	0.2161	59%
Adding Nishimura et al. J Clin Sleep Med. 2021 (k=6)	1.67	[1.32; 2.12]	< 0.01	0.0391	0.1978	51%
Adding Tan et al. Front Endocrinol (Lausanne). 2019 (k=7)	1.60	[1.29; 1.99]	< 0.01	0.0349	0.1867	47%
Adding Unruh et al. Am J Kidney Dis. 2008 (k=8)	1.65	[1.33; 2.05]	< 0.01	0.0396	0.1991	47%
Adding Warsame et al. Nephrol Dial Transplant. 2022 (k=9)	1.61	[1.33; 1.94]	< 0.01	0.0295	0.1716	41%
Adding Yu et al. PLoS One. 2018 (k=10)	1.57	[1.32; 1.88]	< 0.01	0.0268	0.1636	38%
Adding Zhang et al. Front Neurosci. 2022 (k=11)	1.55	[1.31; 1.84]	< 0.01	0.0236	0.1535	33%
**Random effects model**	**1.55**	**[1.31; 1.84]**	**< 0.01**	**0.0236**	**0.1535**	**33%**

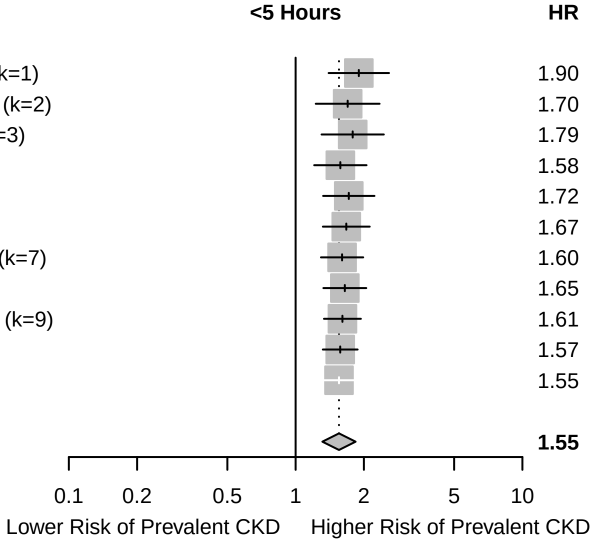

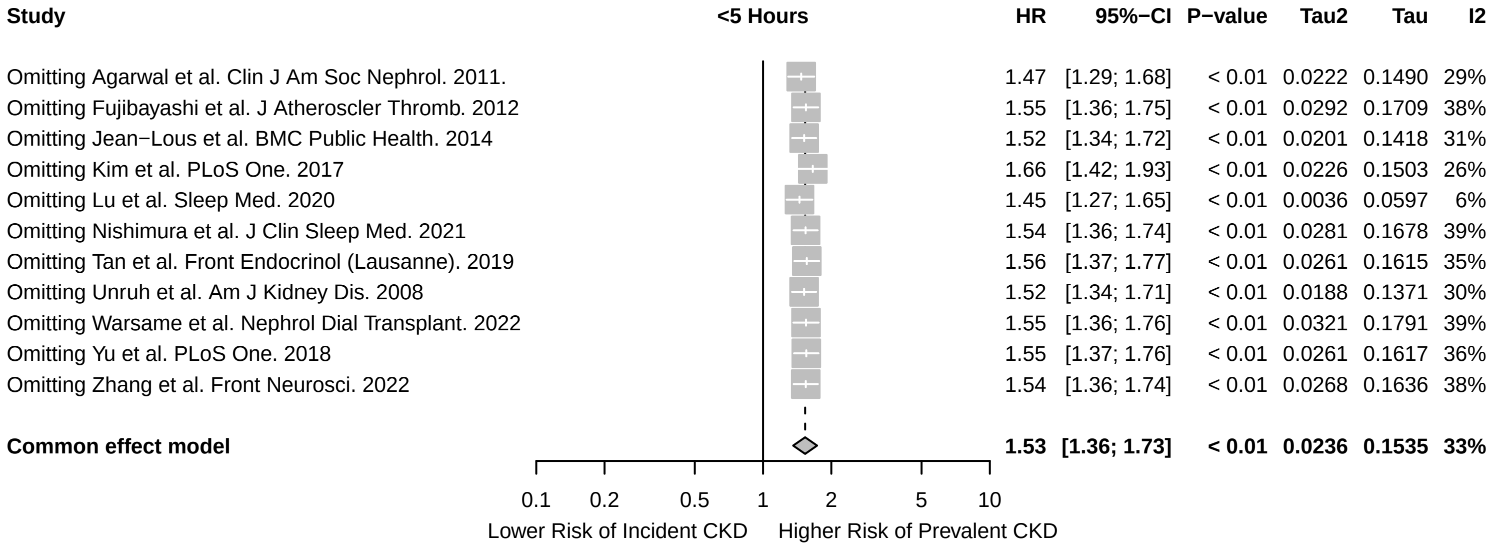

Study
<5 Hours
HR
95%−CI
P−value
Tau2
Tau
I2
Omitting Agarwal et al. Clin J Am Soc Nephrol. 2011. 1.47 [1.29; 1.68] < 0.01 0.0222 0.1490 29%
Omitting Fujibayashi et al. J Atheroscler Thromb. 2012 1.55 [1.36; 1.75] < 0.01 0.0292 0.1709 38%
Omitting Jean−Lous et al. BMC Public Health. 2014 1.52 [1.34; 1.72] < 0.01 0.0201 0.1418 31%
Omitting Kim et al. PLoS One. 2017 1.66 [1.42; 1.93] < 0.01 0.0226 0.1503 26%
Omitting Lu et al. Sleep Med. 2020 1.45 [1.27; 1.65] < 0.01 0.0036 0.0597 6%
Omitting Nishimura et al. J Clin Sleep Med. 2021 1.54 [1.36; 1.74] < 0.01 0.0281 0.1678 39%
Omitting Tan et al. Front Endocrinol (Lausanne). 2019 1.56 [1.37; 1.77] < 0.01 0.0261 0.1615 35%
Omitting Unruh et al. Am J Kidney Dis. 2008 1.52 [1.34; 1.71] < 0.01 0.0188 0.1371 30%
Omitting Warsame et al. Nephrol Dial Transplant. 2022 1.55 [1.36; 1.76] < 0.01 0.0321 0.1791 39%
Omitting Yu et al. PLoS One. 2018 1.55 [1.37; 1.76] < 0.01 0.0261 0.1617 36%
Omitting Zhang et al. Front Neurosci. 2022 1.54 [1.36; 1.74] < 0.01 0.0268 0.1636 38%
Common effect model 1.53 [1.36; 1.73] < 0.01 0.0236 0.1535 33%
0.1
0.2
0.5
1
2
5
10
Lower Risk of Incident CKD
Higher Risk of Prevalent CKD

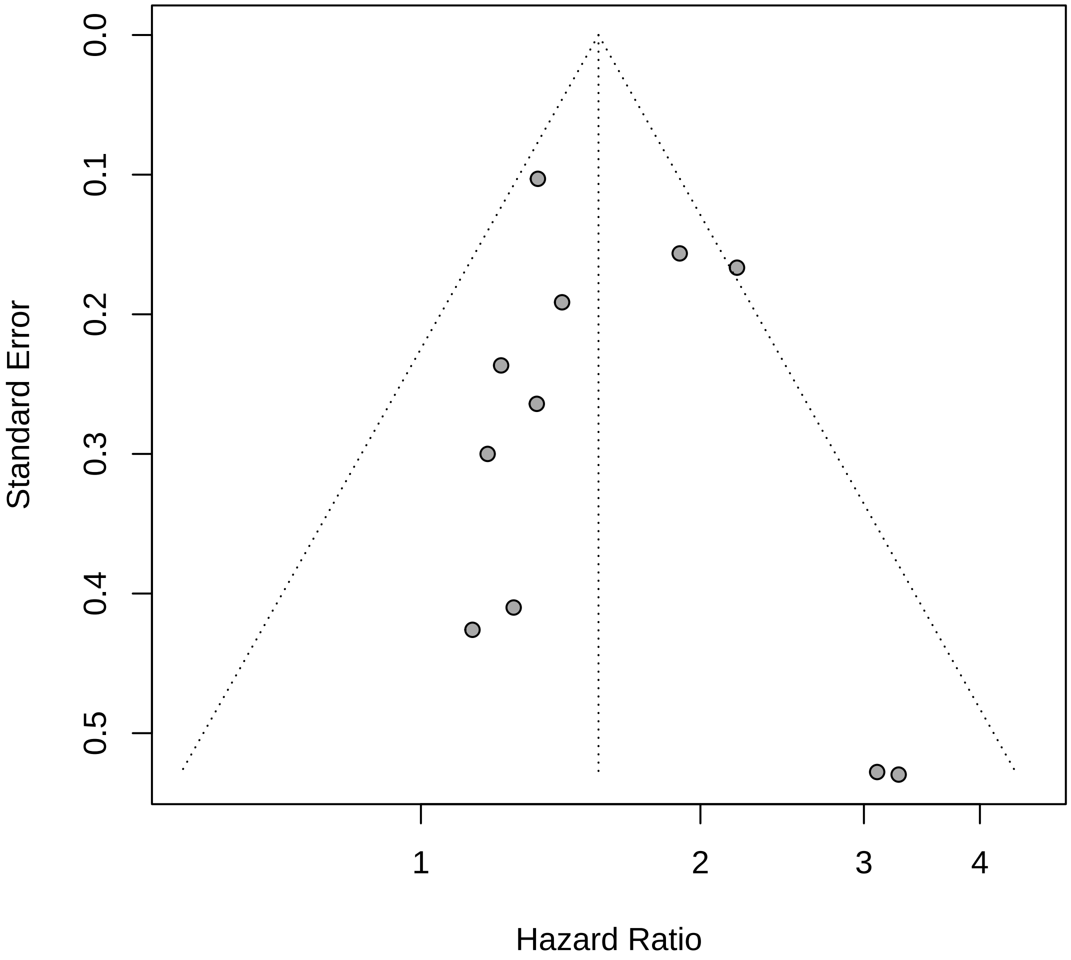

Standard Error
0.0
0.1
0.2
0.3
0.4
0.5
1
2
3
4
Hazard Ratio

Study	HR	95%−CI	P−value	Tau2	Tau	I2
Adding Agarwal et al. Clin J Am Soc Nephrol. 2011. (k=1)	1.90	[1.40; 2.58]	< 0.01	.	.	.
Adding Chang et al. Am J Nephrol. 2019 (k=2)	1.51	[0.83; 2.76]	0.18	0.1210	0.3479	59%
Adding Choi et al. Korean J Intern Med. 2017 (k=3)	1.55	[1.05; 2.28]	0.03	0.0406	0.2014	30%
Adding Fujibayashi et al. J Atheroscler Thromb. 2012 (k=4)	1.55	[1.18; 2.03]	< 0.01	0.0117	0.1080	13%
Adding Jean−Lous et al. BMC Public Health. 2014 (k=5)	1.61	[1.30; 2.00]	< 0.01	0	0	0%
Adding Kim et al. PLoS One. 2017 (k=6)	1.61	[1.39; 1.86]	< 0.01	0	0	0%
Adding Li et al. Clin J Am Soc Nephrol. 2017. (k=7)	1.51	[1.30; 1.75]	< 0.01	0.0044	0.0664	10%
Adding Lu et al. Sleep Med. 2020 (k=8)	1.50	[1.33; 1.70]	< 0.01	0	0	0%
Adding Nishimura et al. J Clin Sleep Med. 2021 (k=9)	1.50	[1.33; 1.69]	< 0.01	0	0	0%
Adding Platinga et al. Am J Kidney Dis. 2011 (k=10)	1.48	[1.32; 1.65]	< 0.01	0	0	0%
Adding Salifu et al. Cardiorenal Med. 2014 (k=11)	1.48	[1.33; 1.65]	< 0.01	0	0	0%
Adding Tan et al. Front Endocrinol (Lausanne). 2019 (k=12)	1.47	[1.32; 1.63]	< 0.01	0	0	0%
Adding Unruh et al. Am J Kidney Dis. 2008 (k=13)	1.48	[1.33; 1.64]	< 0.01	0	0	0%
Adding Warsame et al. Nephrol Dial Transplant. 2022 (k=14)	1.48	[1.34; 1.63]	< 0.01	0	0	0%
Adding Yu et al. PLoS One. 2018 (k=15)	1.39	[1.24; 1.56]	< 0.01	0.0121	0.1099	27%
Adding Zhang et al. Front Neurosci. 2022 (k=16)	1.38	[1.24; 1.54]	< 0.01	0.0100	0.1002	22%
**Random effects model**	**1.38**	**[1.24; 1.54]**	**< 0.01**	**0.0100**	**0.1002**	**22%**

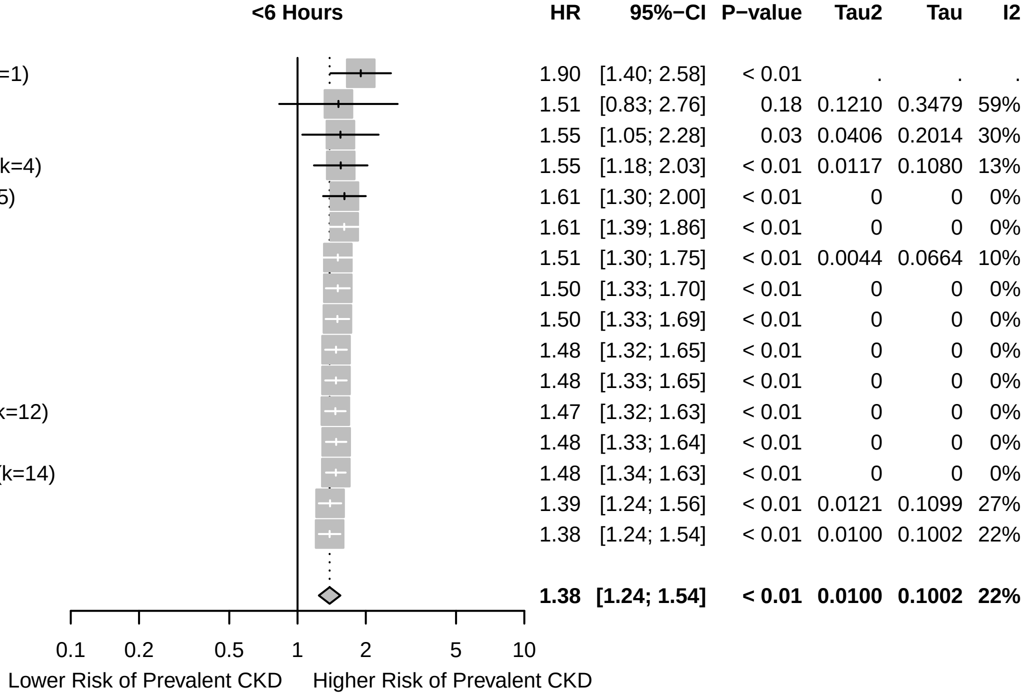

Study	HR	95%−CI	P−value	Tau2	Tau	I2
Omitting Agarwal et al. Clin J Am Soc Nephrol. 2011.	1.33	[1.22; 1.46]	< 0.01	0.0016	0.0398	4%
Omitting Chang et al. Am J Nephrol. 2019	1.38	[1.26; 1.51]	< 0.01	0.0109	0.1045	25%
Omitting Choi et al. Korean J Intern Med. 2017	1.37	[1.26; 1.50]	< 0.01	0.0125	0.1119	27%
Omitting Fujibayashi et al. J Atheroscler Thromb. 2012	1.37	[1.26; 1.50]	< 0.01	0.0128	0.1132	27%
Omitting Jean−Lous et al. BMC Public Health. 2014	1.37	[1.25; 1.49]	< 0.01	0.0115	0.1074	25%
Omitting Kim et al. PLoS One. 2017	1.32	[1.20; 1.46]	< 0.01	0.0073	0.0856	15%
Omitting Li et al. Clin J Am Soc Nephrol. 2017.	1.39	[1.27; 1.53]	< 0.01	0.0108	0.1039	23%
Omitting Lu et al. Sleep Med. 2020	1.37	[1.24; 1.50]	< 0.01	0.0135	0.1164	27%
Omitting Nishimura et al. J Clin Sleep Med. 2021	1.38	[1.26; 1.50]	< 0.01	0.0125	0.1116	27%
Omitting Platinga et al. Am J Kidney Dis. 2011	1.37	[1.25; 1.51]	< 0.01	0.0140	0.1184	27%
Omitting Salifu et al. Cardiorenal Med. 2014	1.37	[1.25; 1.50]	< 0.01	0.0126	0.1121	27%
Omitting Tan et al. Front Endocrinol (Lausanne). 2019	1.37	[1.25; 1.51]	< 0.01	0.0133	0.1155	27%
Omitting Unruh et al. Am J Kidney Dis. 2008	1.36	[1.25; 1.49]	< 0.01	0.0061	0.0783	16%
Omitting Warsame et al. Nephrol Dial Transplant. 2022	1.37	[1.25; 1.50]	< 0.01	0.0132	0.1149	27%
Omitting Yu et al. PLoS One. 2018	1.47	[1.33; 1.62]	< 0.01	0	0	0%
Omitting Zhang et al. Front Neurosci. 2022	1.38	[1.26; 1.51]	< 0.01	0.0121	0.1099	27%
**Common effect model**	**1.37**	**[1.26; 1.50]**	**< 0.01**	**0.0100**	**0.1002**	**22%**

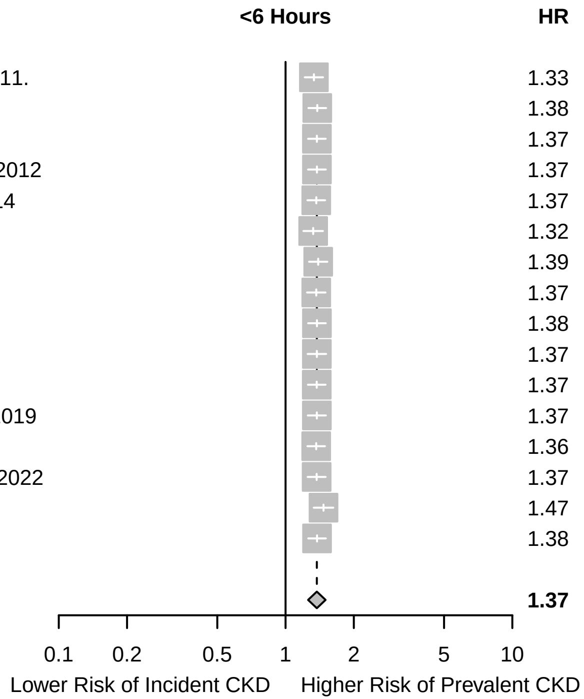

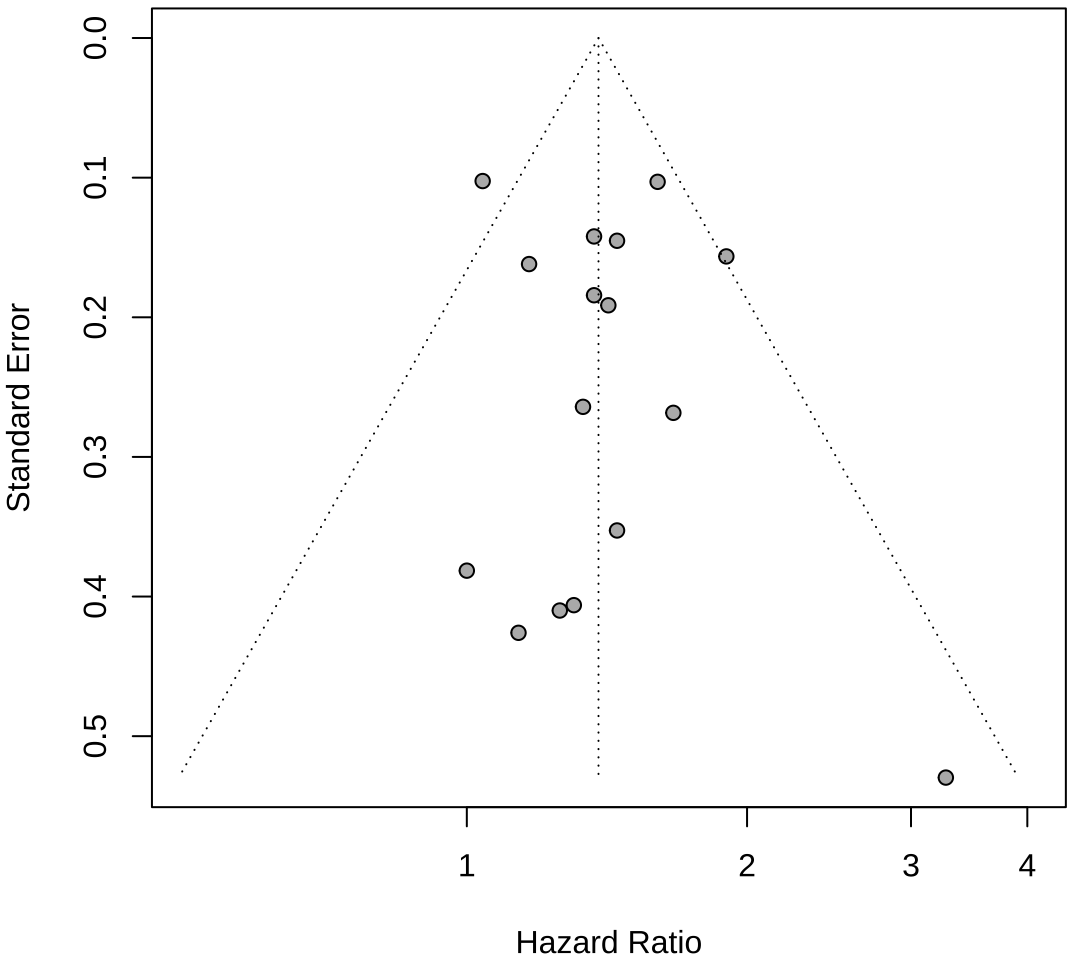
Standard Error
0.0
0.1
0.2
0.3
0.4
0.5
1
2
3
4
Hazard Ratio

Study	<7 Hours	HR	95%−CI	P−value	Tau2	Tau	I2
Adding Agarwal et al. Clin J Am Soc Nephrol. 2011. (k=1)		1.90	[1.40; 2.58]	< 0.01	.	.	.
Adding Chang et al. Am J Nephrol. 2019 (k=2)		1.51	[0.83; 2.76]	0.18	0.1210	0.3479	59%
Adding Choi et al. Korean J Intern Med. 2017 (k=3)		1.37	[0.87; 2.16]	0.17	0.0929	0.3048	58%
Adding Fujibayashi et al. J Atheroscler Thromb. 2012 (k=4)		1.40	[1.02; 1.94]	0.04	0.0435	0.2085	41%
Adding Jean−Lous et al. BMC Public Health. 2014 (k=5)		1.49	[1.16; 1.90]	< 0.01	0.0183	0.1352	22%
Adding Kim et al. PLoS One. 2017 (k=6)		1.37	[1.11; 1.70]	< 0.01	0.0250	0.1581	38%
Adding Li et al. Clin J Am Soc Nephrol. 2017. (k=7)		1.30	[1.07; 1.58]	< 0.01	0.0277	0.1666	44%
Adding Lu et al. Sleep Med. 2020 (k=8)		1.30	[1.09; 1.56]	< 0.01	0.0216	0.1470	35%
Adding Nishimura et al. J Clin Sleep Med. 2021 (k=9)		1.30	[1.10; 1.53]	< 0.01	0.0154	0.1240	26%
Adding Platinga et al. Am J Kidney Dis. 2011 (k=10)		1.31	[1.14; 1.50]	< 0.01	0.0086	0.0925	18%
Adding Salifu et al. Cardiorenal Med. 2014 (k=11)		1.31	[1.15; 1.48]	< 0.01	0.0045	0.0674	10%
Adding Tan et al. Front Endocrinol (Lausanne). 2019 (k=12)		1.31	[1.17; 1.46]	< 0.01	0.0007	0.0259	2%
Adding Unruh et al. Am J Kidney Dis. 2008 (k=13)		1.33	[1.18; 1.51]	< 0.01	0.0076	0.0874	15%
Adding Warsame et al. Nephrol Dial Transplant. 2022 (k=14)		1.34	[1.19; 1.50]	< 0.01	0.0041	0.0643	9%
Adding Yu et al. PLoS One. 2018 (k=15)		1.29	[1.15; 1.45]	< 0.01	0.0120	0.1098	25%
Adding Zhang et al. Front Neurosci. 2022 (k=16)		1.29	[1.15; 1.44]	< 0.01	0.0095	0.0976	20%
**Random effects model**		**1.29**	**[1.15; 1.44]**	**< 0.01**	**0.0095**	**0.0976**	**20%**

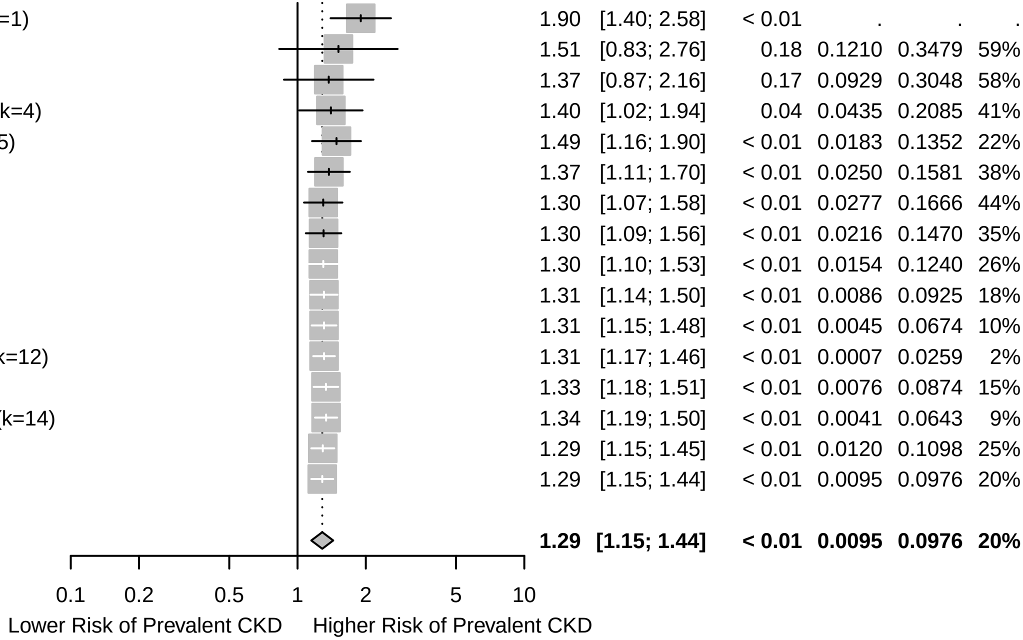

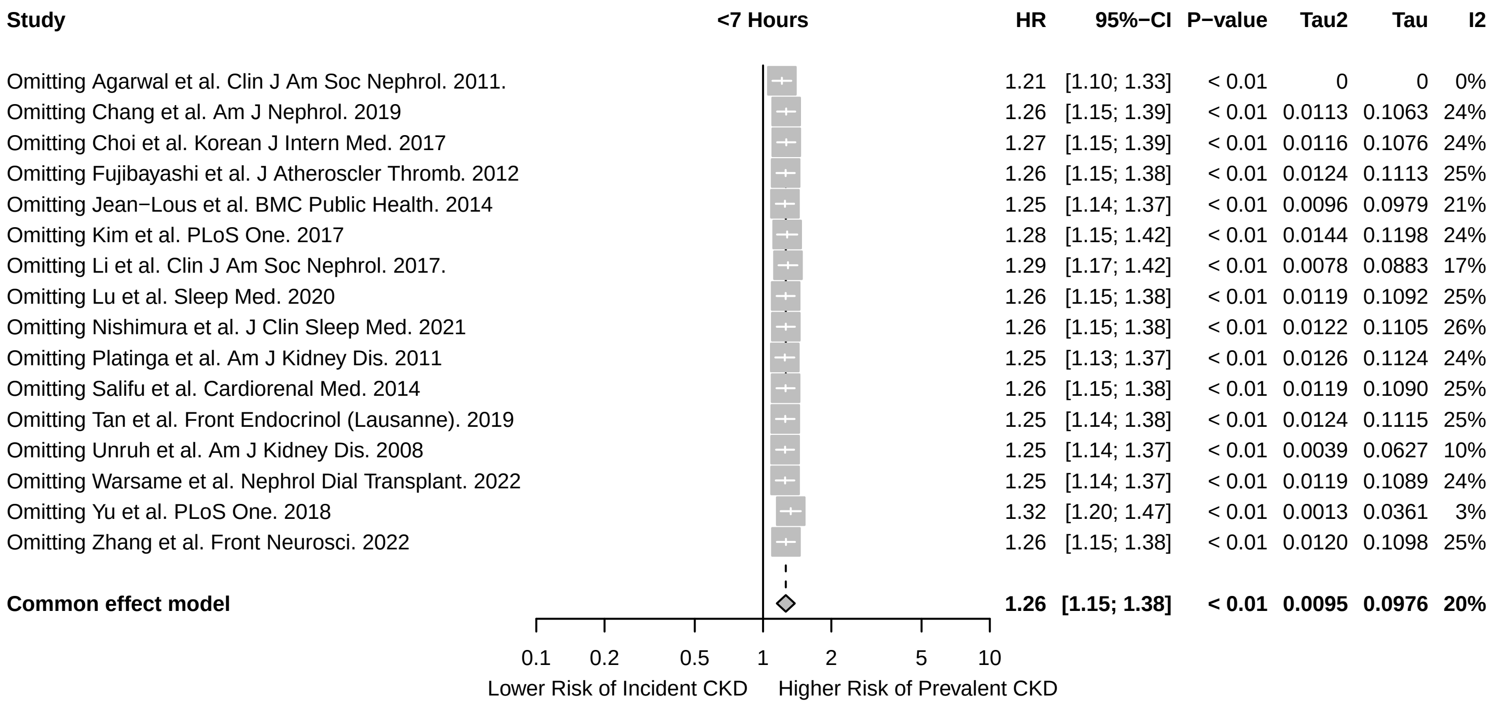
Study
<7 Hours
HR
95%−CI
P−value
Tau2
Tau
I2
Omitting Agarwal et al. Clin J Am Soc Nephrol. 2011. 1.21 [1.10; 1.33] < 0.01 0 0 0%
Omitting Chang et al. Am J Nephrol. 2019 1.26 [1.15; 1.39] < 0.01 0.0113 0.1063 24%
Omitting Choi et al. Korean J Intern Med. 2017 1.27 [1.15; 1.39] < 0.01 0.0116 0.1076 24%
Omitting Fujibayashi et al. J Atheroscler Thromb. 2012 1.26 [1.15; 1.38] < 0.01 0.0124 0.1113 25%
Omitting Jean−Lous et al. BMC Public Health. 2014 1.25 [1.14; 1.37] < 0.01 0.0096 0.0979 21%
Omitting Kim et al. PLoS One. 2017 1.28 [1.15; 1.42] < 0.01 0.0144 0.1198 24%
Omitting Li et al. Clin J Am Soc Nephrol. 2017. 1.29 [1.17; 1.42] < 0.01 0.0078 0.0883 17%
Omitting Lu et al. Sleep Med. 2020 1.26 [1.15; 1.38] < 0.01 0.0119 0.1092 25%
Omitting Nishimura et al. J Clin Sleep Med. 2021 1.26 [1.15; 1.38] < 0.01 0.0122 0.1105 26%
Omitting Platinga et al. Am J Kidney Dis. 2011 1.25 [1.13; 1.37] < 0.01 0.0126 0.1124 24%
Omitting Salifu et al. Cardiorenal Med. 2014 1.26 [1.15; 1.38] < 0.01 0.0119 0.1090 25%
Omitting Tan et al. Front Endocrinol (Lausanne). 2019 1.25 [1.14; 1.38] < 0.01 0.0124 0.1115 25%
Omitting Unruh et al. Am J Kidney Dis. 2008 1.25 [1.14; 1.37] < 0.01 0.0039 0.0627 10%
Omitting Warsame et al. Nephrol Dial Transplant. 2022 1.25 [1.14; 1.37] < 0.01 0.0119 0.1089 24%
Omitting Yu et al. PLoS One. 2018 1.32 [1.20; 1.47] < 0.01 0.0013 0.0361 3%
Omitting Zhang et al. Front Neurosci. 2022 1.26 [1.15; 1.38] < 0.01 0.0120 0.1098 25%
Common effect model 1.26 [1.15; 1.38] < 0.01 0.0095 0.0976 20%
0.1
0.2
0.5
1
2
5
10
Lower Risk of Incident CKD
Higher Risk of Prevalent CKD

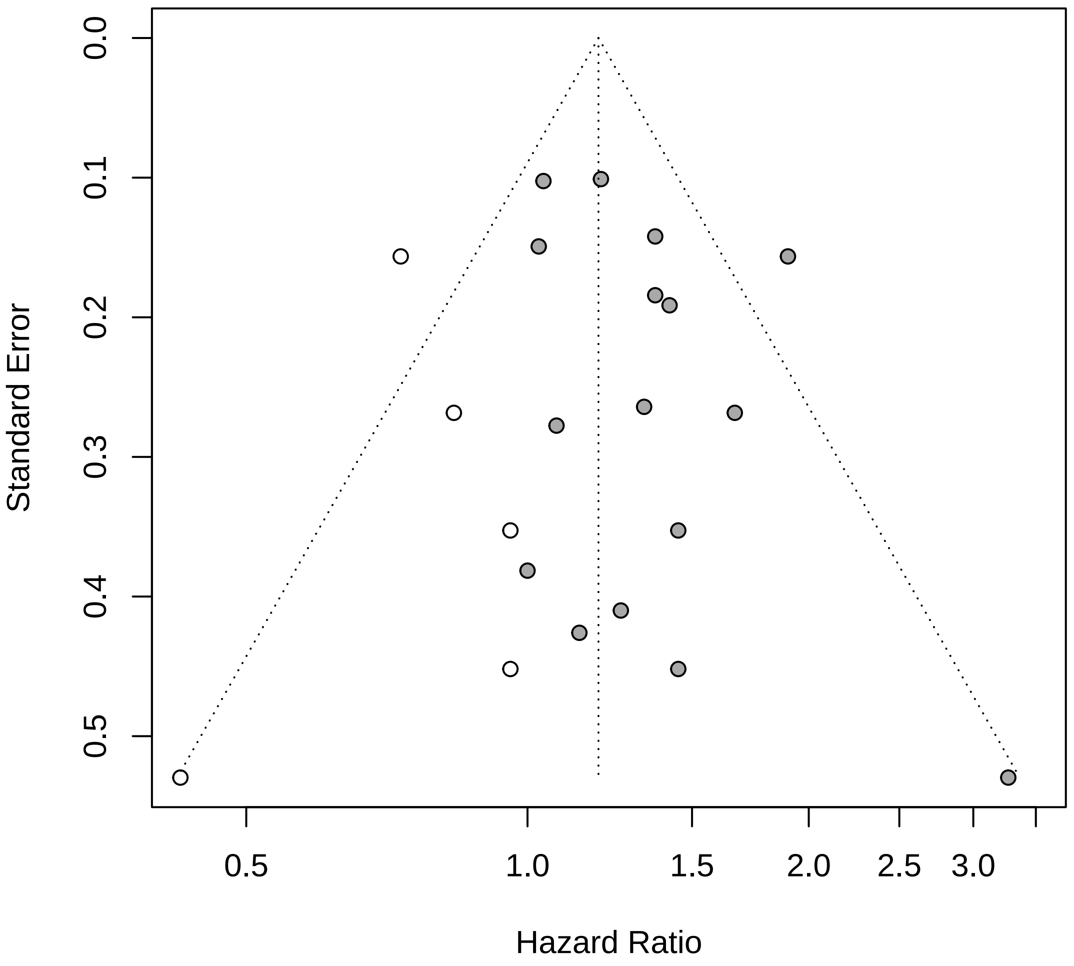

Standard Error
0.0
0.1
0.2
0.3
0.4
0.5
0.5
1.0
1.5
2.0
2.5
3.0
Hazard Ratio

Study	HR	95%−CI	P−value	Tau2	Tau	I2
Adding Chang et al. Am J Nephrol. 2019 (k=1)	1.86	[0.91; 3.82]	0.09	.	.	.
Adding Choi et al. Korean J Intern Med. 2017 (k=2)	1.51	[1.10; 2.07]	0.01	0	0	0%
Adding Kim et al. PLoS One. 2017 (k=3)	1.72	[1.38; 2.14]	< 0.01	0	0	0%
Adding Li et al. Clin J Am Soc Nephrol. 2017. (k=4)	1.62	[1.34; 1.95]	< 0.01	0	0	0%
Adding Lu et al. Sleep Med. 2020 (k=5)	1.55	[1.31; 1.83]	< 0.01	0	0	0%
Adding Platinga et al. Am J Kidney Dis. 2011 (k=6)	1.48	[1.27; 1.73]	< 0.01	0.0021	0.0459	5%
Adding Salifu et al. Cardiorenal Med. 2014 (k=7)	1.49	[1.29; 1.73]	< 0.01	0	0	0%
Adding Tan et al. Front Endocrinol (Lausanne). 2019 (k=8)	1.53	[1.32; 1.77]	< 0.01	0.0019	0.0433	4%
**Random effects model**	**1.53**	**[1.32; 1.77]**	**< 0.01**	**0.0019**	**0.0433**	**4%**

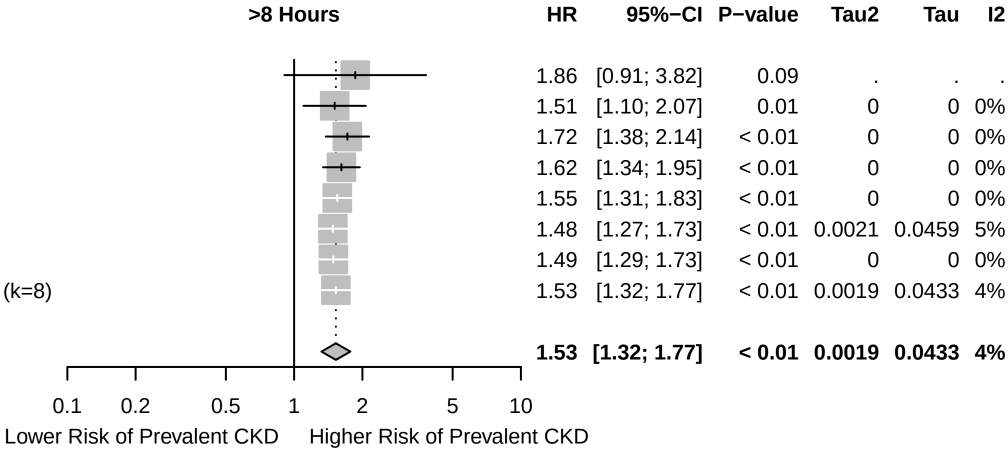

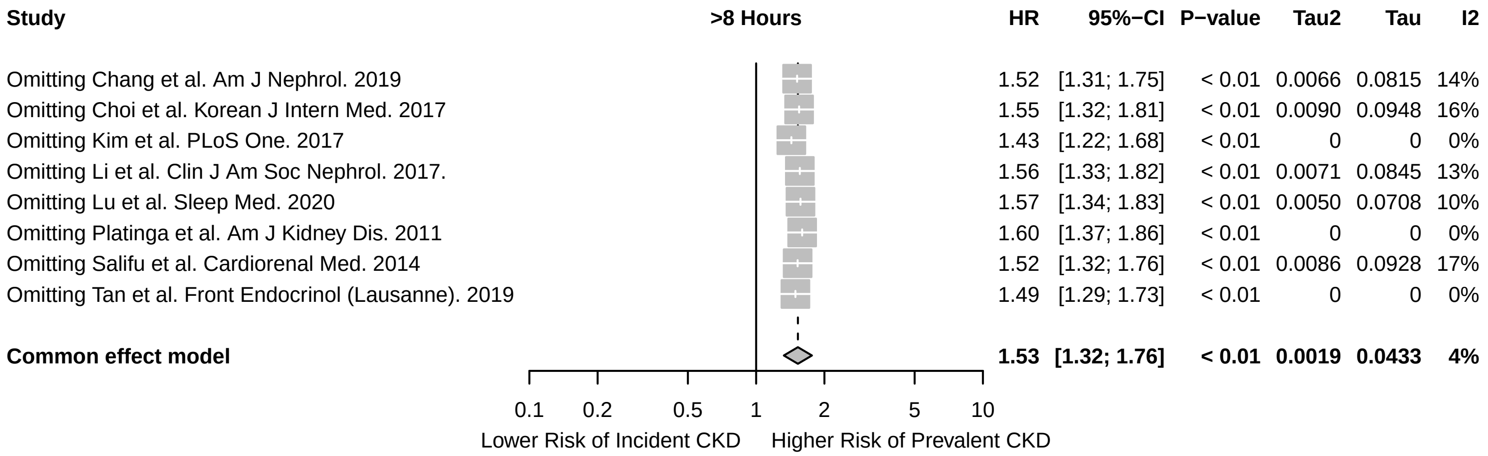
Study
>8 Hours
HR
95%−CI
P−value
Tau2
Tau
I2
Omitting Chang et al. Am J Nephrol. 2019 1.52 [1.31; 1.75] < 0.01 0.0066 0.0815 14%
Omitting Choi et al. Korean J Intern Med. 2017 1.55 [1.32; 1.81] < 0.01 0.0090 0.0948 16%
Omitting Kim et al. PLoS One. 2017 1.43 [1.22; 1.68] < 0.01 0 0 0%
Omitting Li et al. Clin J Am Soc Nephrol. 2017. 1.56 [1.33; 1.82] < 0.01 0.0071 0.0845 13%
Omitting Lu et al. Sleep Med. 2020 1.57 [1.34; 1.83] < 0.01 0.0050 0.0708 10%
Omitting Platinga et al. Am J Kidney Dis. 2011 1.60 [1.37; 1.86] < 0.01 0 0 0%
Omitting Salifu et al. Cardiorenal Med. 2014 1.52 [1.32; 1.76] < 0.01 0.0086 0.0928 17%
Omitting Tan et al. Front Endocrinol (Lausanne). 2019 1.49 [1.29; 1.73] < 0.01 0 0 0%
Common effect model 1.53 [1.32; 1.76] < 0.01 0.0019 0.0433 4%
0.1
0.2
0.5
1
2
5
10
Lower Risk of Incident CKD
Higher Risk of Prevalent CKD

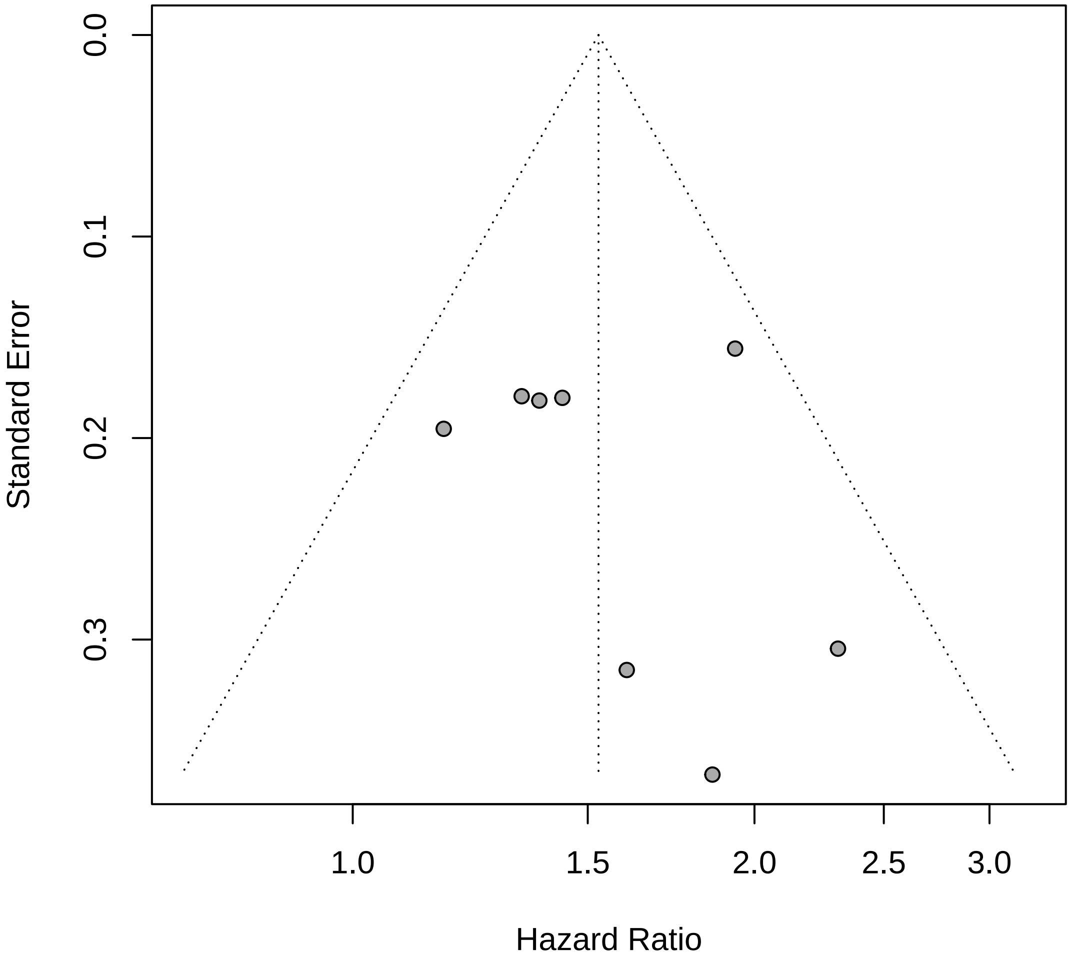

0.0
0.1
0.2
0.3
Standard Error
1.0
1.5
2.0
2.5
3.0
Hazard Ratio

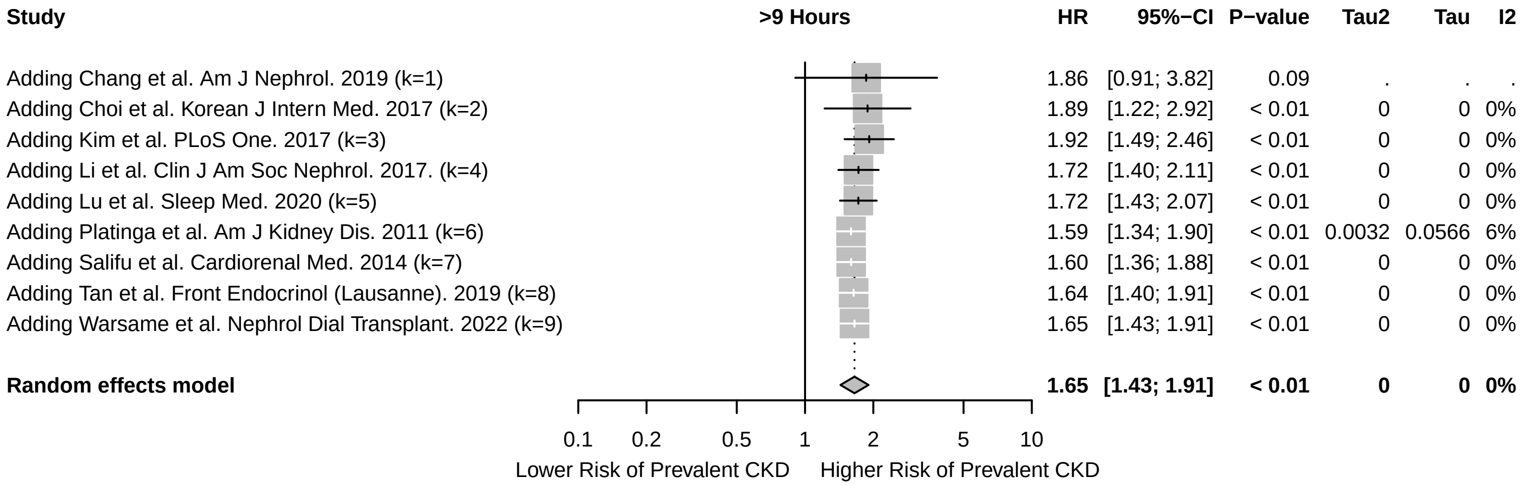
Study
>9 Hours
HR
95%−CI
P−value
Tau2
Tau
I2
Adding Chang et al. Am J Nephrol. 2019 (k=1) 1.86 [0.91; 3.82] 0.09 . . .
Adding Choi et al. Korean J Intern Med. 2017 (k=2) 1.89 [1.22; 2.92] < 0.01 0 0 0%
Adding Kim et al. PLoS One. 2017 (k=3) 1.92 [1.49; 2.46] < 0.01 0 0 0%
Adding Li et al. Clin J Am Soc Nephrol. 2017. (k=4) 1.72 [1.40; 2.11] < 0.01 0 0 0%
Adding Lu et al. Sleep Med. 2020 (k=5) 1.72 [1.43; 2.07] < 0.01 0 0 0%
Adding Platinga et al. Am J Kidney Dis. 2011 (k=6) 1.59 [1.34; 1.90] < 0.01 0.0032 0.0566 6%
Adding Salifu et al. Cardiorenal Med. 2014 (k=7) 1.60 [1.36; 1.88] < 0.01 0 0 0%
Adding Tan et al. Front Endocrinol (Lausanne). 2019 (k=8) 1.64 [1.40; 1.91] < 0.01 0 0 0%
Adding Warsame et al. Nephrol Dial Transplant. 2022 (k=9) 1.65 [1.43; 1.91] < 0.01 0 0 0%
Random effects model 1.65 [1.43; 1.91] < 0.01 0 0 0%
0.1
0.2
0.5
1
2
5
10
Lower Risk of Prevalent CKD
Higher Risk of Prevalent CKD

Study	>9 Hours	HR	95%−CI	P−value	Tau2	Tau	I2
Omitting Chang et al. Am J Nephrol. 2019		1.64	[1.42; 1.90]	< 0.01	0	0	0%
Omitting Choi et al. Korean J Intern Med. 2017		1.64	[1.41; 1.90]	< 0.01	0	0	0%
Omitting Kim et al. PLoS One. 2017		1.58	[1.35; 1.86]	< 0.01	0	0	0%
Omitting Li et al. Clin J Am Soc Nephrol. 2017.		1.71	[1.46; 2.00]	< 0.01	0	0	0%
Omitting Lu et al. Sleep Med. 2020		1.65	[1.42; 1.91]	< 0.01	0	0	0%
Omitting Platinga et al. Am J Kidney Dis. 2011		1.75	[1.50; 2.04]	< 0.01	0	0	0%
Omitting Salifu et al. Cardiorenal Med. 2014		1.66	[1.43; 1.92]	< 0.01	0	0	0%
Omitting Tan et al. Front Endocrinol (Lausanne). 2019		1.62	[1.40; 1.88]	< 0.01	0	0	0%
Omitting Warsame et al. Nephrol Dial Transplant. 2022		1.64	[1.40; 1.91]	< 0.01	0	0	0%
**Common effect model**		**1.65**	**[1.43; 1.91]**	**< 0.01**	**0**	**0**	**0%**

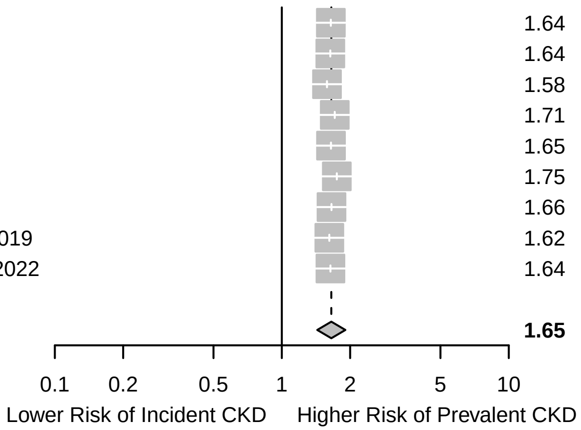

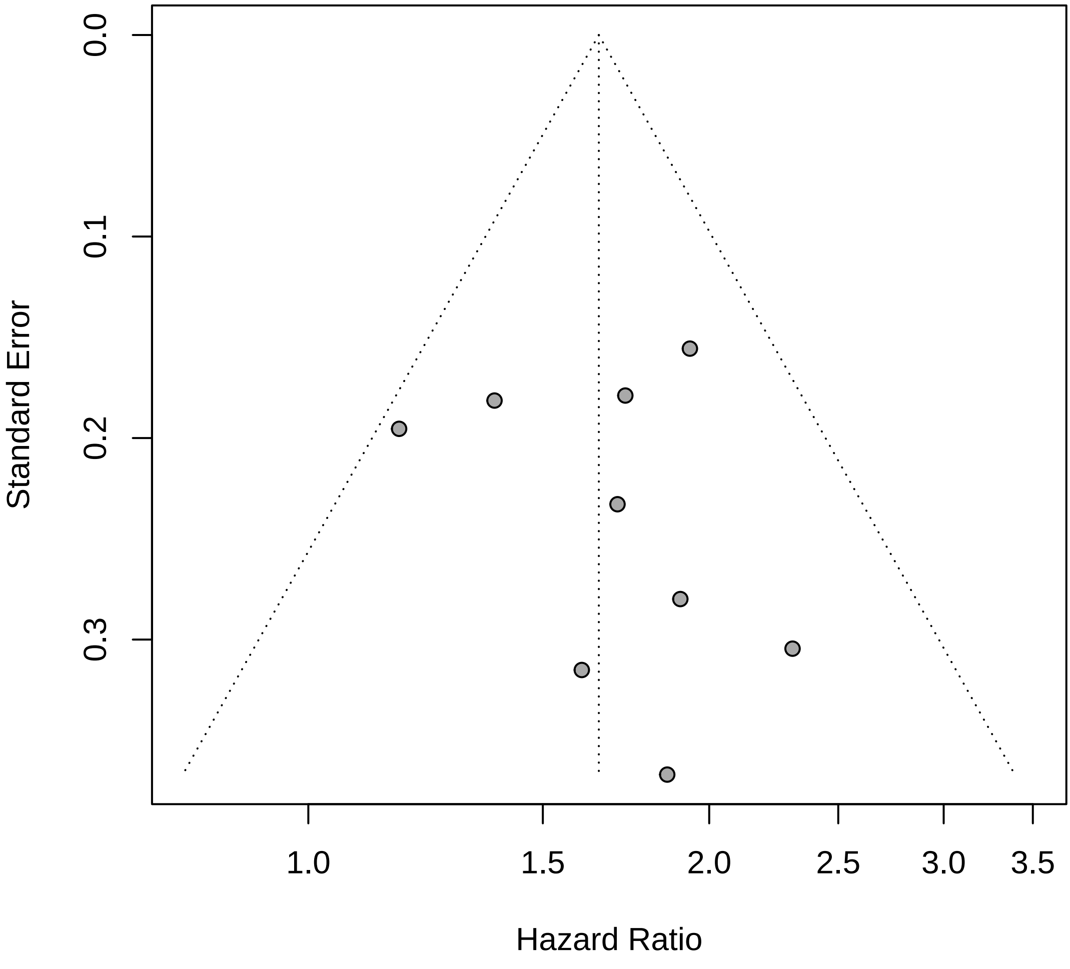

0.0
0.1
0.2
0.3
Standard Error
1.0
1.5
2.0
2.5
3.0
3.5
Hazard Ratio
