# Supplementary material for: The association of sleep duration with the risk of chronic kidney disease: a systematic review and meta-analysis
Source: Clin Kidney J. 2024 Jul 11;17(8):sfae177. doi: 10.1093/ckj/sfae177 (PMC11304598; doi:10.1093/ckj/sfae177)
Supplement: sfae177_Supplemental_Files [file sfae177_supplemental_files.zip › S8. Subgroup Analyses.docx]

Supplement 8. Subgroup Analyses for Prevalent CKD

| Outcome | Studies | RR (95% CI) | *I^2^* (%) | 95% PI | *p-*value |
| --- | --- | --- | --- | --- | --- |
| **<4 Hours** |  |  |  |  |  |
| Methodology |  |  |  |  |  |
| Cohort Study | 2 | 1.34 (0.91 to 1.97) | 56 | NR | 0.83 |
| Cross-Sectional Study | 6 | 1.41 (1.23 to 1.61) | 5 | 1.12 to 1.76 |  |
| Mode of Measurement of Sleep Duration and Quality |  |  |  |  |  |
| Self-Reported Questionnaire | 6 | 1.31 (1.13 to 1.53) | 15 | 0.91 to 1.20 | 0.19 |
| Polysomnography | 2 | 1.10 (1.03 to 1.18) | 66 | 0.79 to 1.89 |  |
|  |  |  |  |  |  |
| **<5 Hours** |  |  |  |  |  |
| Methodology |  |  |  |  |  |
| Cohort Study | 3 | 1.56 (1.11 to 2.20) | 72 | NR | 0.97 |
| Cross-Sectional Study | 6 | 1.58 (1.16 to 2.14) | 63 | 0.60 to 4.11 |  |
| Mode of Measurement of Sleep Duration and Quality |  |  |  |  |  |
| Self-Reported Questionnaire | 8 | 1.47 (1.22 to 1.78) | 32 | 0.96 to 2.25 | 0.59 |
| Polysomnography | 2 | 1.91 (0.76 to 4.84) | 51 | NR |  |
|  |  |  |  |  |  |
| **<6 Hours** |  |  |  |  |  |
| Methodology |  |  |  |  |  |
| Cohort Study | 2 | 1.25 (0.79 to 1.99) | 75 | NR | 0.74 |
| Cross-Sectional Study | 8 | 1.36 (1.20 to 1.55) | 58 | 0.96 to 1.93 |  |
| Mode of Measurement of Sleep Duration and Quality |  |  |  |  |  |
| Self-Reported Questionnaire | 13 | 1.32 91.21 to 1.46) | 0 | 1.19 to 1.47 | 0.44 |
| Polysomnography | 2 | 1.91 (0.76 to 4.84) | 51 | NR |  |
|  |  |  |  |  |  |
| **<7 Hours** |  |  |  |  |  |
| Methodology |  |  |  |  |  |
| Cohort Study | 2 | 1.07 (0.90 to 1.27) | 64 | NR | 0.06 |
| Cross-Sectional Study | 8 | 1.32 (1.14 to 1.52) | 66 | 0.88 to 1.97 |  |
| Mode of Measurement of Sleep Duration and Quality |  |  |  |  |  |
| Self-Reported Questionnaire | 13 | 1.20 (1.09 to 1.32) | 0 | 1.08 to 1.34 | 0.33 |
| Polysomnography | 2 | 1.91 (0.76 to 3.40) | 51 | NR |  |
| Actigraphy | 1 | 1.90 (1.40 to 2.58) | NR | NR |  |
|  |  |  |  |  |  |
| **>8 Hours** |  |  |  |  |  |
| Methodology |  |  |  |  |  |
| Cohort Study | 2 | 1.93 (1.64 to 2.27) | 0 | NR | **<0.01** |
| Cross-Sectional Study | 6 | 1.41 (1.26 to 1.59) | 66 | 0.99 to 2.02 |  |

CI, confidence interval; PI, prediction interval; RR, risk ratio
