# Supplementary material for: The association of sleep duration with the risk of chronic kidney disease: a systematic review and meta-analysis
Source: Clin Kidney J. 2024 Jul 11;17(8):sfae177. doi: 10.1093/ckj/sfae177 (PMC11304598; doi:10.1093/ckj/sfae177)
Supplement: sfae177_Supplemental_Files [file sfae177_supplemental_files.zip › S9. GRADE.pdf]

Supplement 9. Evaluation of quality of pooled evidence using the Grading of Recommendations Assessment, Development and Evaluation (GRADE) framework.

Outcomes	Effect size (95% CI)	Number of patients (number of included studies)	I^2	A	B	C	D	E	F	G	H	Quality of evidence
Incident CKD												
≤4 Hours	1.41 (1.16 to 1.71)	298,707 (6)	55			-1				+1		Moderate
≤5 Hours	1.46 (1.22 to 1.76)	394,067 (10)	63			-1						Low
≤6 Hours	1.18 (1.09 to 1.29)	426,518 (12)	13									Moderate
≤7 Hours	1.19 (1.12 to 1.28)	445,734 (15)	8									Moderate
≥8 Hours	1.15 (1.03 to 1.28)	435,475 (14)	46			-1						Low
≥9 Hours	1.46 (1.28 to 1.68)	151,977 (8)	7									Moderate
PSQI	0.91 (0.65 to 1.26)	1329 (5)	49			-1						Low
Prevalent CKD												
≤4 Hours	1.33 (1.13 to 1.56)	289,738 (9)	19							+1		Moderate
≤5 Hours	1.54 (1.31 to 1.81)	1,606,044 (7)	27									Moderate
≤6 Hours	1.39 (1.24 to 1.56)	1,753,574 (20)	27									Moderate
≤7 Hours	1.29 (1.15 to 1.44)	1,753,574 (10)	20									Moderate
≥8 Hours	1.53 (1.32 to 1.77)	1,609,613 (8)	4			-1						Low
≥9 Hours	1.65 (1.43 to 1.91)	1,196,343 (3)	0									Low

A: risk of bias among included studies. B: imprecision. C: inconsistency. D: indirectness of evidence. E: publication bias. F: dose response gradient. G: large effect size. H: biases increasing confidence in the estimate.
