# Supplementary figures and images for: The association of sleep duration with the risk of chronic kidney disease: a systematic review and meta-analysis
Source: Clin Kidney J. 2024 Jul 11;17(8):sfae177. doi: 10.1093/ckj/sfae177 (PMC11304598; doi:10.1093/ckj/sfae177)

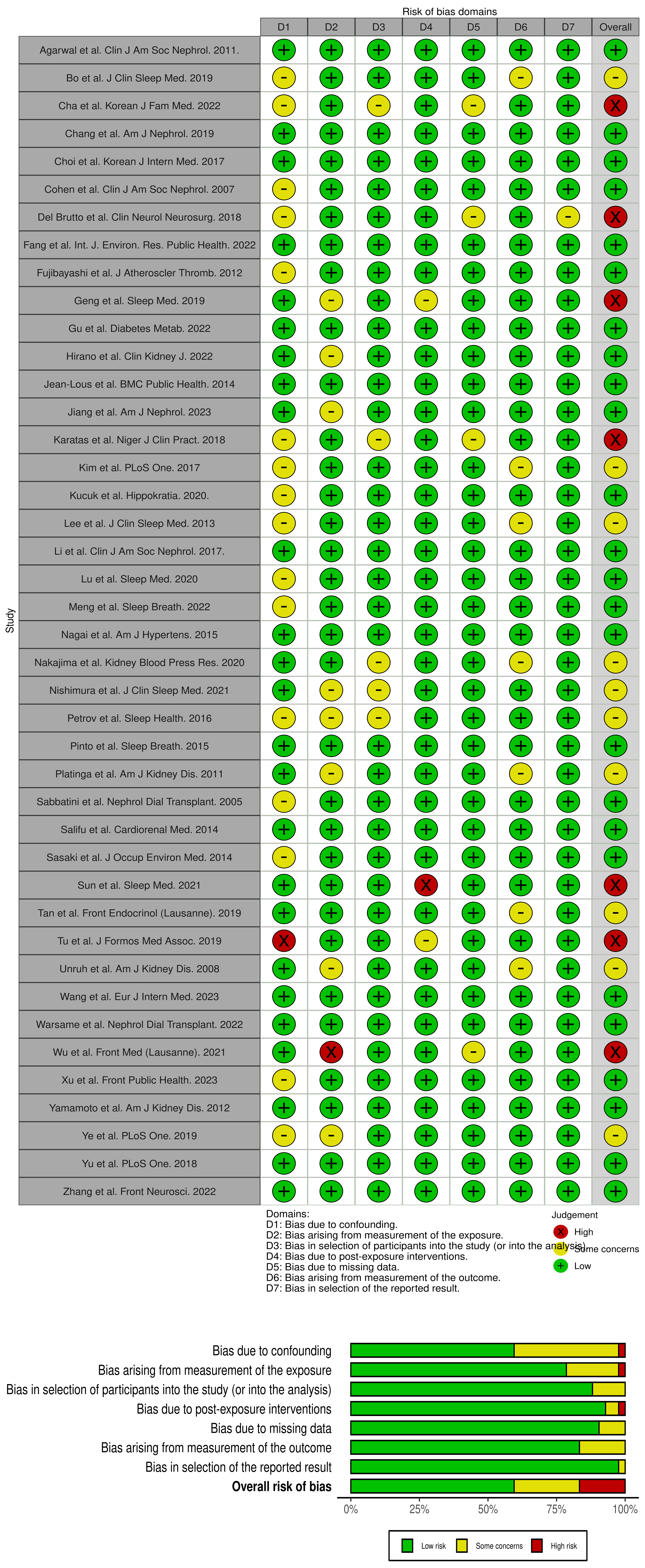

Supplement: sfae177_Supplemental_Files [file sfae177_supplemental_files.zip › S2. ROB.tif]

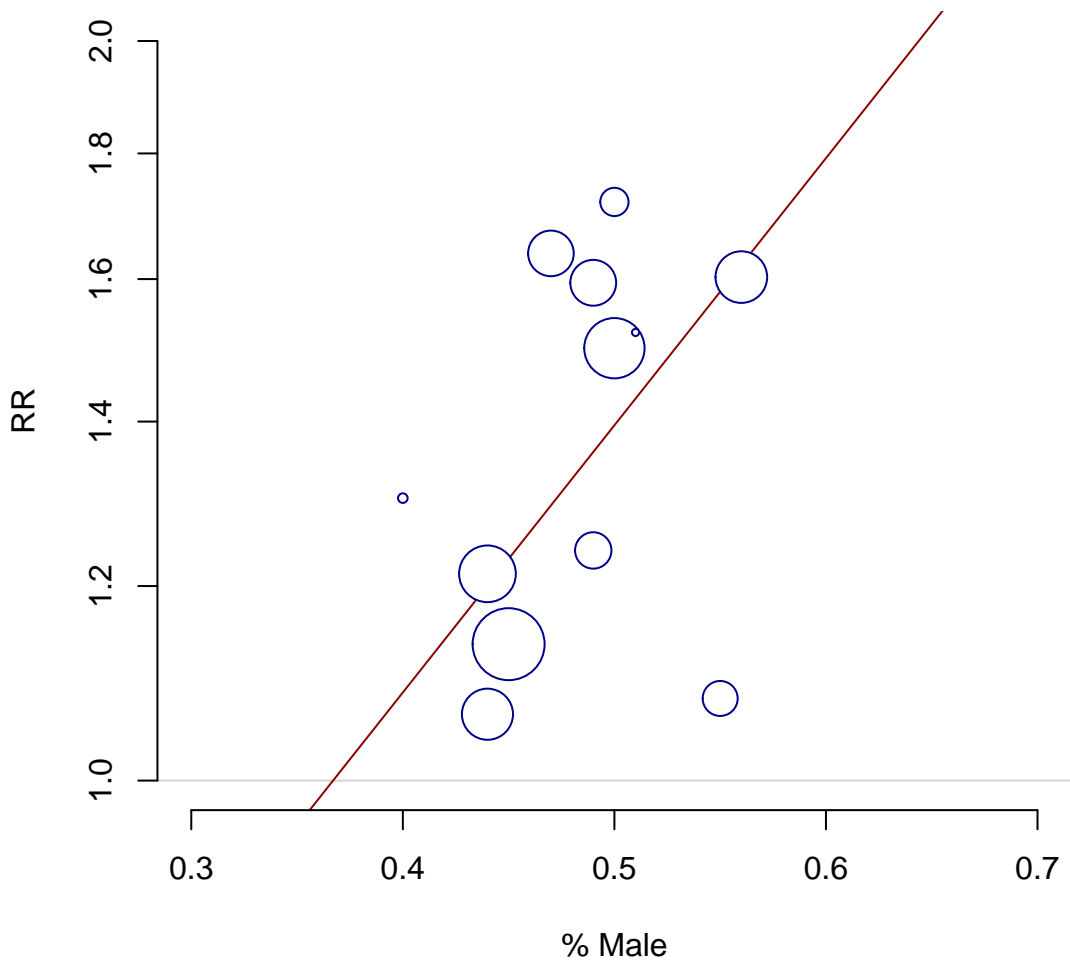

Supplement: sfae177_Supplemental_Files [file sfae177_supplemental_files.zip › S3. Bubble Plot for 4 Hours.pdf]
